# Effect of the Microstructure of the Semiconductor Support on the Photocatalytic Performance of the Pt-PtO*_x_*/TiO_2_ Catalyst System

**DOI:** 10.3390/ma14040943

**Published:** 2021-02-17

**Authors:** Katalin Majrik, Zoltán Pászti, László Korecz, Judith Mihály, Zoltán May, Péter Németh, Catia Cannilla, Giuseppe Bonura, Francesco Frusteri, András Tompos, Emília Tálas

**Affiliations:** 1 Research Centre for Natural Sciences, Institute of Materials and Environmental Chemistry, Eötvös Loránd Research Network (ELKH), H-1117 Budapest, Magyar Tudósok Körútja 2, Hungary; majrik.katalin@ttk.hu (K.M.); korecz.laszlo@ttk.hu (L.K.); mihaly.judith@ttk.hu (J.M.); may.zoltan@ttk.hu (Z.M.); nemeth.peter@ttk.hu (P.N.); tompos.andras@ttk.mta.hu (A.T.); talas.emilia@ttk.hu (E.T.); 2 Department of Earth and Environmental Sciences, University of Pannonia, H-8200 Veszprém, Egyetem út 10, Hungary; 3 National Council of Research–CNR-ITAE, ‘‘Nicola Giordano’’, Via S. Lucia 5, 98126 Messina, Italy; cannilla@itae.cnr.it (C.C.); giuseppe.bonura@itae.cnr.it (G.B.); francesco.frusteri@cnr.it (F.F.)

**Keywords:** sol–gel method, calcination, high-temperature H_2_ treatment, TEM, XPS, ESR

## Abstract

The influence of the semiconductor microstructure on the photocatalytic behavior of Pt-PtO*_x_*/TiO_2_ catalysts was studied by comparing the methanol-reforming performance of systems based on commercial P25 or TiO_2_ from sol–gel synthesis calcined at different temperatures. The Pt co-catalyst was deposited by incipient wetness and formed either by calcination or high-temperature H_2_ treatment. Structural features of the photocatalysts were established by X-ray powder diffraction (XRD), electron spin resonance (ESR), X-ray photoelectron spectroscopy (XPS), optical absorption, Raman spectroscopy and TEM measurements. In situ reduction of Pt during the photocatalytic reaction was generally observed. The P25-based samples showed the best H_2_ production, while the activity of all sol–gel-based samples was similar in spite of the varying microstructures resulting from the different preparation conditions. Accordingly, the sol–gel-based TiO_2_ has a fundamental structural feature interfering with its photocatalytic performance, which could not be improved by annealing in the 400–500 °C range even by scarifying specific surface area at higher temperatures.

## 1. Introduction

Recently, a great deal of work has been devoted to photocatalytic hydrogen production [1,2]. Since hydrogen is regarded as an important secondary energy source for the future [3,4,5]; photocatalytic implementation of hydrogen production would give an opportunity to transform solar energy into chemical energy for storage [6]. Large numbers of publications have indicated that photoinduced reforming of alcohols such as methanol, glycerol and more, over semiconducting oxides in the presence of water, could be an efficient way of hydrogen generation [7,8,9,10,11,12,13]. Even if the conversion of the methanol in the photoinduced reforming reaction (1) is rather low compared to other methanol-reforming systems, reaction (1) is widely studied and used as a model reaction to compare photocatalysts [14,15].

(1) $${\mathrm{CH}}_{3}{\mathrm{OH}\;+\;H}_{2}O\;\overset{\mathrm{Photocatalyst},\;\mathrm{hv}}{\underset{\;}{⇌}}{\;\mathrm{CO}}_{2}{+3\;H}_{2}$$

The overvoltage of H_2_ evolution and the recombination rate of the photoexcited electron-hole pairs can be lowered by depositing noble metal particles onto the surface of the semiconductor [16,17]. Accordingly, the structure of the semiconductor, the nature of the co-catalyst, and the interaction between them simultaneously influence the activity of these photocatalysts [18].

TiO_2_ is a widely used photocatalyst because of its stability and activity in a wide range of reactions. TiO_2_ applications can be divided into two main categories—“energy” and “environmental” [19]. The popularity of TiO_2_ is also due to its non-toxic nature, its relatively low cost and its availability in large amounts. It should also be noted that several different ways exist to prepare TiO_2_, providing opportunities to produce materials tailored for diverse applications [20,21,22,23]. In order to obtain TiO_2_ nanostructures, the most frequently used techniques are sol–gel, micelle and inverse micelle, sol, hydrothermal, solvothermal, direct oxidation, chemical vapor deposition, physical vapor deposition, electrodeposition, sonochemical, microwave methods [19].

The sol–gel process is considered as one of the most promising approaches due to its numerous advantages such as excellent chemical homogeneity, nanosized crystallized structures of high purity at relatively low temperature and no need for expensive particular apparatus [24,25,26]. It gives the possibility to obtain doped and co-doped TiO_2_ and TiO_2_-based composite materials [25]. As a result, a huge amount of recipes of sol–gel TiO_2_ prepared with very different parameters can be found in the literature. The common feature is that the preparation of TiO_2_ by the sol–gel method involves the formation of a metal-oxo-polymer network from molecular precursors such as metal salts or very often metal alkoxides [24]. The sol–gel process is influenced by several factors such as the reactivity of metal alkoxides, pH of the reaction medium, reaction temperature, nature of the solvent and the additive or the water to alkoxide ratio [24]. Hydrolysis and condensation governed by these factors are the two main reactions that take place under the sol–gel procedure; their mutual interplay determines the product formation. In order to obtain small colloidal clusters that lead to a well-controlled, uniform particle size, the use of bulky, branched alkoxy groups (such as isopropoxides) is favorable because they result in reduced hydrolysis- and condensation rates [27]. Additives (electrolytes, polymers, complexing agents, acid or base catalysts) can also modulate the relative rate of the condensation reactions [24].

In general, samples obtained in the first step of the sol-gel preparation procedure are amorphous. For instance, the as-derived precipitates obtained by sol–gel hydrolysis precipitation of titanium isopropoxide (Ti(OC_3_H_7_)_4_) starting material without special additives are hydrous oxides in amorphous phase [24,28]; the sample made of TiCl_4_ with ethanol is almost amorphous after drying at 100 °C [29]. Disregarding some exceptions [30,31], in order to induce crystallization, the sol–gel derived samples have to been further treated thermally by hydrothermal procedure or calcination [24].

If the calcination is chosen, one of the most important issues will be its temperature, a key parameter to control not only the content of impurities [24,32] and crystallinity but particle size and surface area, too [24,29,33].

It is not surprising that titania precipitates obtained from metal–organic precursors, in the presence of organic modifiers, can contain carbonaceous remnants. Based on thermogravimetric measurements, it has been reported that heat treatment at temperatures higher than 400 °C [32] is necessary to remove these carbonaceous species.

The presence of an amorphous part is believed to be unfavorable for photocatalysis [34,35] because the disordered phase is known to contain trap sites for the recombination of photo-excited electrons and holes in TiO_2_ [35]. According to Reference 24, the amorphous gel prepared without special additives from titanium-isopropoxide begins to crystallize upon heat treatment at 200 °C, giving a mixture of amorphous titania and nanocrystalline anatase TiO_2_; a complete transformation to crystalline anatase TiO_2_ requires calcination at least at 450 °C [24]. Similarly, in another study, the amorphous content was reported to decrease gradually from 83% to 0% when the temperature of calcination was increased from 200 °C up to 500 °C [29].

According to Ikeda et al. “highly active TiO_2_ photocatalyst should simultaneously possess two properties: A large surface area to adsorb a substrate and a high degree of crystallinity (or few surface and bulk defects) to reduce e^−^–h^+^ recombination” [36]. The high temperature treatment, which usually improves the crystallinity of TiO_2_, can induce the aggregation of small nanoparticles and decrease the surface area. For example, in the case of preparation of TiO_2_-anatase thin films by the sol–gel method, the highest conversion in the photocatalytic probe reaction was obtained with samples calcined at 450 °C for 1 h, the same conditions for obtaining the best compromise between the surface area and crystallinity [21]. In order to increase both crystallinity and specific surface area (SSA), several sophisticated methods have been developed such as the sol–gel method combined with the two-step calcining process [37].

Another limiting factor in increasing the calcination temperature is the appearance of the rutile phase. Literature suggests that this transformation is not characteristic up to 500 °C [21,24,28,29,33,38] in case of un-doped TiO_2_, although doping with metals can decrease the transformation temperature [39,40].

Considering all factors, it appears that, in most cases, sol–gel-prepared TiO_2_ materials require a calcination treatment for adequate photocatalytic performance and the optimal temperature of this preparation step is in the 400–500 °C range.

Regarding the co-catalysts, it appears that Pt is the most effective metallic element, followed by Pd, for photoreforming catalysts [8]. In order to load platinum nanoparticles on the surface of the semiconductors, the available methods include deposition of pre-prepared metal colloids [41], precipitation [42] and most commonly photodeposition [42,43] or impregnation [42]. The impregnated salt can be further transformed into metal or metal oxide nanoparticles by annealing [8]. For this co-catalyst formation, either treatment at elevated temperature in hydrogen/inert gas or calcination in air can be chosen. Depending on the platinum precursor and the method of co-catalyst formation, Pt nanoparticles with different oxidation states are loaded on the surface of the TiO_2_ [44]. Literature indicates that in the photocatalytic reforming reactions over Pt/TiO_2_ catalysts, the metallic state of platinum is the most active because metallic Pt is a better catalyst for proton reduction and H_2_ formation than partially oxidized Pt [45]. However, it cannot be ruled out that ionic Pt species also contribute to the photocatalytic reaction [46,47,48]. As far as the amount of Pt is concerned, literature results suggest that the metal loading has an optimal value below 1 weight% [9,10].

Although the Pt-PtO*_x_*/TiO_2_ photocatalyst system is the subject of ongoing scientific interest and general conclusions about the role of the nature of the semiconductor and its defect structure in the photocatalytic process are established, there are still many details that deserve further clarification. For example, the versatility of the sol–gel-based methods renders them very popular among the TiO_2_ semiconductor preparation routes, while their predominantly low-temperature nature may result in certain fundamental features concerning the microstructure of the semiconductor. It is continuously investigated how these structural features can limit the photocatalytic performance of the material, and whether they can be mitigated by an appropriate co-catalyst deposition approach. A related issue is how the formation and the interaction of the Pt co-catalyst with the semiconductor are influenced by the structural features of the TiO_2_ support and how this influence is reflected in the photocatalytic properties. In order to obtain insight into these details at a modeling level, in this study Pt-PtO*_x_*/TiO_2_ photocatalysts supported on titania prepared either by flame synthesis or by sol–gel precipitation in the presence of citric acid [49] followed by calcination at varying temperatures and activated after Pt loading by either high-temperature hydrogen treatment or calcination in air were compared in the photoinduced reforming of methanol. The observations were interpreted in terms of the structural and electronic properties of the TiO_2_ and the chemical state of the platinum co-catalyst, with emphasis on the state of the catalyst under the working conditions, which was approached by comparing the physical–chemical characterization results of the fresh and used samples.

## 2. Materials and Methods

### 2.1. Materials

Titania nanoparticles were synthesized using titanium-isopropoxide (≥97.0%% Sigma Aldrich Inc., St. Louis, MO, USA) Citric acid (ACS reagent, ≥99.5%) for gel formation was obtained from Sigma Aldrich Inc. Pt(NH_3_)_4_(NO_3_)_2_ (≥50.0% Pt basis, Sigma-Aldrich Inc.) was the precursor of supported Pt nanoparticles. Other chemicals included methanol (a.r., 99.996%, Molar Chemicals Ltd., Budapest, Hungary), absolute ethanol (100.0%, AnalaR NORMAPUR, VWR International, Fontenay-sous-Bois, France) and hydrochloric acid (36,4%, AnalaR NORMAPUR, VWR), hydrofluoric acid (38 41%, a.r., Reanal, Budapest, Hungary) and nitric acid (65 w%, a.r., Molar Chemicals Ltd.). Double distilled water (18 MΩ) was used for the synthesis of photocatalysts and for the preparation of methanol solution. The gases (H_2_, N_2,_ Ar) used in this work were products of Linde Gáz Magyarország Zrt. (Budapest, Hungary) with 5.0 purity. Special mixture of 5% H_2_ in N_2_ for calibration of the gas chromatograph was bought from Messer Hungarogáz Ltd. (Budapest, Hungary).

### 2.2. Synthesis of Photocatalysts

The TiO_2_ samples were prepared by the sol–gel method as described before [50]. Briefly, titanium-isopropoxide was stirred with citric acid and absolute ethanol for 180 min at room temperature and then heated at 65 °C until gel formation. The gel was dried overnight at 80–90 °C under ambient conditions then calcined in air for 5 h. As we attempted to avoid both the presence of a large amount of amorphous phase and the drastic decrease of the specific surface area, calcination temperatures of 400 °C, 450 °C and 500 °C were selected in accordance with the literature and our previous experience. Sol–gel prepared TiO_2_ samples were obtained by high yield. For comparison, the flame-synthesized Aerolyst^®^ TiO_2_ (P25, Evonik, Essen, Germany) was also used as semiconductor.

Introduction of Pt was performed from aqueous solution of the Pt(NH_3_)_4_(NO_3_)_2_ precursor by incipient wetness impregnation. The 0.7 weight% Pt loading used in this work corresponds to the upper limit of the optimum range [9] but still facilitates investigations by photoelectron spectroscopy. Samples were dried at 80–90 °C overnight under ambient conditions. The dried samples were either reduced for 1 h at 400 °C in H_2_ atmosphere or calcined in air for 1 h at 300 °C. It may be noted that small alterations of the synthesis procedure such as the use of ethanol in the sol–gel procedure and introduction of Pt onto the TiO_2_ could be beneficial from a sustainability standpoint [51].

**Figure 1:**
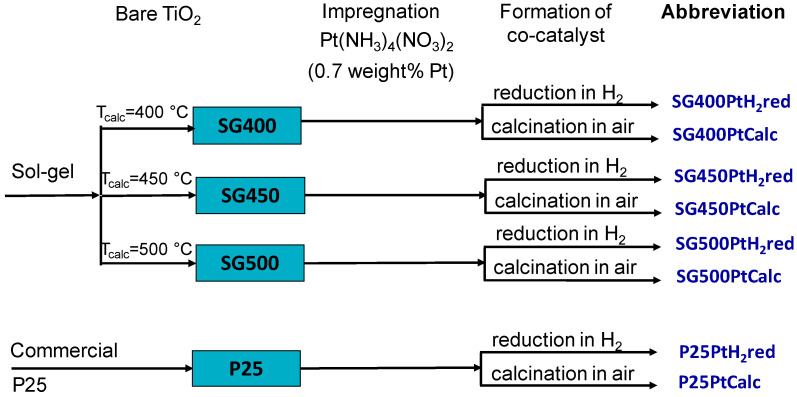
Denomination of the various Pt-PtO*_x_*/TiO_2_ catalysts.

The lineage and denomination of the various PtO*_x_*/TiO_2_ catalysts are depicted in Figure 1. The Pt content of the photocatalysts was checked by inductively coupled plasma-optical emission spectrometry (ICP-OES) technique after microwave-assisted dissolution in 1:8 mixture of concentrated nitric acid and hydrofluoric acid. For the measurements, a simultaneous SPECTRO GENESIS instrument (SPECTRO Analytical Instruments GmbH, Kleve, Germany) with axial plasma observation was used.

### 2.3. Physical–Chemical Characterization of Photocatalysts

BET specific surfaces were measured by recording nitrogen physisorption isotherms in volumetric equipment (ASDI RXM 100, Advanced Scientific Design Inc. Bamco-Surplus, Texas City, TX, USA). Sample pretreatment involved annealing in inert gas flow at 200 °C for 1.5 h then evacuation prior to cooling to the temperature of liquid nitrogen.

X-ray powder diffraction (XRD) patterns were measured by a Philips model PW 3710-based PW 1050 Bragg-Brentano (Philips, Eindhoven, The Netherlands) parafocusing goniometer using CuKα radiation (λ = 0.15418 nm) equipped with graphite monochromator and proportional counter. Silicon powder (NIST SRM 640, National Institute of Standards and Technology, Gaithersburg, MD, USA) served as an internal standard and the XRD patterns were evaluated by profile fitting. Reference cards from the ICDD PDF-2 (1998) database were used. Crystallite sizes were calculated from the broadening of the reflection peaks according to the Scherrer equation.

Optical properties of the samples were investigated by diffuse reflectance UV-visible spectroscopy using a Jasco V-570 UV-VIS spectrophotometer (Jasco, Tokyo, Japan) equipped with an NV-470 integrating sphere. The data were collected between 800 and 200 nm wavelengths with 100 nm/min speed.

The electron spin resonance (ESR) experiments were performed at ambient temperature with a Bruker Elexsys E500 CW X-band spectrometer (microwave frequency ~9.8 GHz, microwave power 1 mW, modulation frequency 100 kHz, modulation amplitude 1 G, Bruker, Rheinstetten, BRD). Calibration of the magnetic field was performed with an NMR field meter of 0.01G precision. Signal intensity, linewidth and g-factor (spectroscopic splitting factor) values were extracted from the measured spectra.

For transmission electron microscopic (TEM) investigation, we used a Philips CM12 (see also the Supplementary Material, Philips, Eindhoven, The Netherlands) and a 200 keV Thalos Thermo Scientific transmission electron microscope (ThermoFisher Scientific, Eindhoven, The Netherlands). A dispersion of the powdered samples prepared in sonicated 2-propanol was deposited dropwise on a holey carbon-coated support grid. In order to investigate the size and distribution of Pt particles, the scanning transmission electron microscopy mode and the energy dispersive X-ray detectors of the 200 keV Talos microscope were used. Fast-Fourier transforms were calculated using the Digital Micrograph 3.6.1 software (Gatan, Pleasanton, CA, USA).

Surface composition and chemical states in the photocatalysts were investigated by X-ray photoelectron spectroscopy (XPS) in an EA125 electron spectrometer manufactured by OMICRON Nanotechnology GmbH (Taunusstein, Germany). Details of the measurements are described in the Supplementary Material Section. High resolution spectra were recorded to identify chemical states of the elements with the help of XPS databases [52,53]. Quantification was performed using CasaXPS [54] and XPSMultiQuant [55,56].

### 2.4. Photocatalytic Hydrogen Generation

The photocatalytic reaction in the UV-visible region was carried out in a cylindrical quartz reactor (140 mm in height and 60 mm in diameter) equipped with magnetic stirrers, gas inputs and outputs under a continuous nitrogen flow of 20 cm^3^/min flow rate. An Osram HQL de luxe 125W lamp (Osram Licht AG, Munich, Germany) was used as light source. The reaction was carried out at room temperature with an initial methanol concentration of 6 v/v% in distilled water. The amount of catalyst and the reaction volume was 0.140 g and 260 cm^3^, respectively. The H_2_ production from the methanol reforming reaction was monitored for 270 min by a gas chromatograph (Agilent 7820A, Agilent Technologies, Santa Clara, CA, USA) equipped with SUPELCO Carboxen 1010 column and TCD detector (Supelco Analytical, Bellefonte, PA, USA). During analysis, argon added to the vapor-gas mixture before the sampling valve was used as an internal standard. The reaction set up is depicted in the supplementary material (Figure S1). The H_2_ production was expressed as H_2_ production rate (mmol h^−1^). Measurements performed on catalysts prepared in different runs as well as repeated measurements on catalysts from the same batch revealed the high reproducibility of the H_2_ production data. Used samples were recovered after the photocatalytic reaction by decantation from the aqueous methanol solution, followed by washing with 3 × 50 cm^3^ absolute ethanol and drying under N_2_ flow.

## 3. Results

### 3.1. Photocatalytic Hydrogen Generation

**Figure 2:**
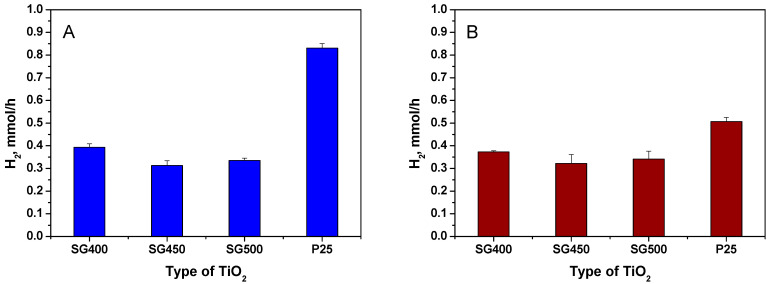
Hydrogen production rate in photocatalytic reforming reaction of methanol. Measured after 270 min; in the case of sol–gel-based samples, H_2_ evolution data are averages of results from two parallel TiO_2_ preparation. (**A**) co-catalyst formation by H_2_ treatment; (**B**) co-catalyst formation by calcination. Error bars are based on measurements repeated two times.

First, we compare hydrogen yields of the various catalysts prepared in this work in photocatalytic methanol-reforming. In Figure 2A, data obtained for catalysts deposited on either the P25 or the various SG supports activated by hydrogen treatment are shown, while Figure 2B summarizes results for the catalysts activated by calcination.

According to blank experiments, hydrogen formation was not observed in dark. This rendered direct comparison of the catalytic properties of the bare TiO_2_ materials impossible in this study. Otherwise, our hydrogen production results are of the same order as those in other works [13,57,58,59]. As can be seen from Figure 2, the P25-based samples gave a higher H_2_ production rate than the sol–gel-based samples, irrespectively if the Pt co-catalyst was formed by hydrogen reduction or calcination. Very surprisingly, essentially no correlation was observed between the pre-annealing temperature of the sol–gel-based supports and the activity of the photocatalysts prepared on them.

Since it is generally accepted that metallic Pt is a key requirement for photocatalytic hydrogen generation in aqueous systems, it is quite plausible that the hydrogen production of the P25-based catalyst activated by calcination is lower than that of the P25PtH_2_red material. On the other hand, it is noteworthy that the sol–gel-based samples with co-catalyst formed by calcination showed essentially the same H_2_ evolution rate (Figure 2B) as their reduced counterparts, in agreement with the findings of our previous works [44,60].

While the superior performance of the P25-based photocatalysts is by no means unexpected, our assumption was that the inherent structural imperfections of the sol–gel titanias should be improved by increasing the calcination temperature of the support which should have a marked effect on the photocatalytic performance. Therefore, detailed structural studies were performed both on the support materials and the photocatalysts in order to answer two questions: (i) why the calcination temperature of the support has a negligible effect on the hydrogen production of the SG-based catalysts and (ii) why catalyst activation by calcination, which obviously deteriorates the H_2_ yield in case of the P25-based catalyst, is not worse or is even slightly more beneficial (as observed in our previous study [60]) than the reductive co-catalyst forming in case of the SG-based materials.

### 3.2. Characterization of the Bare TiO_2_ Materials

In order to establish correlations between the photocatalytic performance of the studied catalysts and the structural features of the support materials, first the TiO_2_ materials before Pt loading were characterized by structure-sensitive methodologies.

The main textural and structural properties of the non-platinized TiO_2_ samples are summarized in Table 1. Specific surface area (SSA) of the sol–gel-prepared samples was in the same range as that of P25; although the highest calcination temperature at SG500 resulted in ~ 40% decrease of SSA and a certain increase in grain size which are consistent with the literature results [24,29,33,38,61]. The critical nuclei size for rutile formation was estimated to be in the range of 40–50 nm [24], thus rutile formation under the conditions applied in this work is not expected.

According to the results of XRD measurements all of our sol–gel-prepared TiO_2_ samples contained exclusively anatase as a crystalline phase while P25 contained anatase and rutile as usual [62]. Raman spectroscopic measurements (see Figure S2 in Supplementary Materials) confirmed the XRD results.

TEM micrographs of the SG TiO_2_ and the P25 materials are presented in the Supplementary Materials (see Figure S3). A characteristic feature of the SG samples is that they consist of small randomly oriented primary crystallites (see Table 1 and Figure S3A–C) which partially coalesce into irregularly shaped larger agglomerates. In contrast, the primary particle size of the P25 material is larger and the agglomeration of the crystallites is less pronounced (Figure S3D). Accordingly, any member of the SG series appears to be less ordered than the P25 material, due to the numerous interfacial regions between the primary crystallites (and possibly the amorphous phase around them, at least in the SG450 and the SG400).

**Figure 3:**
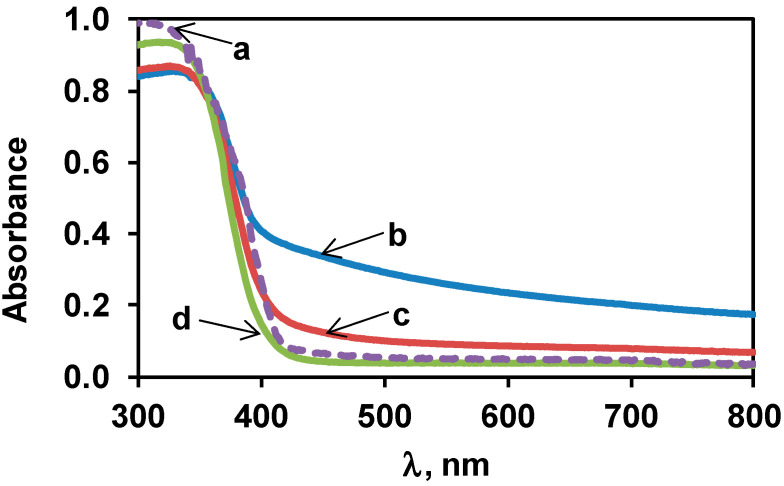
UV-Vis spectra of the bare TiO_2_ samples. Line a: P25; line b: sol–gel-prepared and calcined at 400 °C (SG400), line c: sol–gel-prepared and calcined at 450 °C (SG450), line d: sol–gel-prepared and calcined at 500 °C (SG500).

Diffuse reflectance UV-Visible spectra of the TiO_2_ samples are shown in Figure 3. Adsorption edges became sharper with increasing the temperature of the calcination after the sol–gel precipitation (cf. lines b, c and d in Figure 3). Finally, the adsorption edge of sample SG500 (line d in Figure 3) looked very similar to that of P25 (line a in Figure 3).

A Tauc plot analysis of the UV-Vis spectra with the assumption of indirect transitions estimated the optical bandgap of the TiO_2_ materials to be around 3.0–3.15 eV (see also Section S2.3 and Figure S4 in the Supplementary Materials). In the spectra of the studied samples (especially in the case of the SG400 and the SG450, see Figure 3) a tail region is observable at wavelengths well beyond the fundamental absorption edge. These so-called weak absorption tails in semiconductors indicate the presence of localized electronic states in the bandgap region, which are traditionally connected to structural disorder [63]. Similar long tails have appeared in the absorbance spectra of TiO_2_ nanomaterials prepared by sol–gel-like techniques [64,65] including our previous work [50], or even in the case of well-crystallized anatase particles after surface amorphization by gamma irradiation [66]. The disorder in these systems was attributed to the low preparation temperatures, and, to a smaller extent to the incorporation of oxygen vacancies. It should be taken into account that the amount of the amorphous phase could be significant at lower calcination temperatures [29]. Both the absorbance data in Figure 3 and their semi-logarithmic representation (see Figure S4B in the Supplementary Materials) demonstrate that upon increasing the temperature of the calcination step of the sol–gel preparation, the structural disorder of the obtained TiO_2_ decreases, possibly along with the amount of the amorphous fraction. Nevertheless, even the SG500 material contains significantly more disorder-induced localized electronic states near the valence and conduction band edges than the P25 sample.

It must be noted that even if these disorder-induced electronic states may offer optical absorption well into the visible range, the generated electron-hole pairs remain localized either at or near the surface of the semiconductor or even in its bulk and their chance for participation in a photocatalytic process remains very low. On the other hand, these states can act as electron traps and/or recombination centers, which further diminishes the photocatalytic performance of the material.

**Figure 4:**
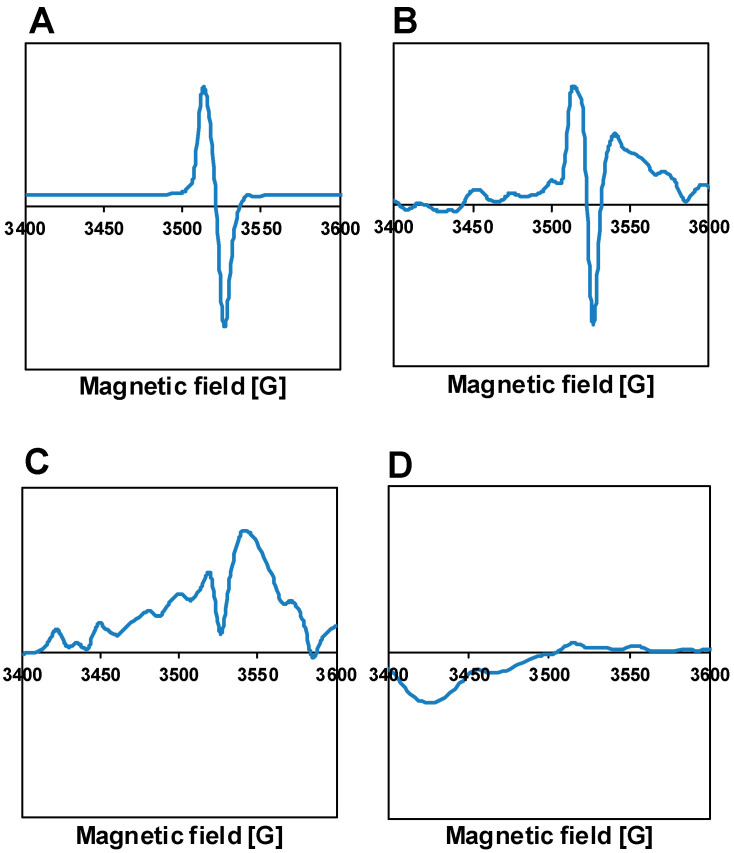
Electron spin resonance (ESR) spectra of the bare TiO_2_ samples; (**A**) sol–gel-prepared, calcined at 400 °C (SG400); (**B)** sol–gel-prepared, calcined at 450 °C (SG450); (**C**) sol–gel-prepared, calcined at 500 °C (SG500); (**D**) P25.

Results of electron spin resonance (ESR) measurements of the sol–gel-based TiO_2_ samples are shown in Figure 4. The spectra are similar to those we obtained in our previous work [60], while P25 is essentially ESR silent. In the SG400 sample, a narrow signal in the vicinity of free electron g factor (g ≈ 2.00) appeared at about 3520 G. Based on literature data, this signal is assigned to a single electron trapped in an oxygen vacancy [60,67], although alternate interpretations are also available. For example, T.S. Rajaraman and et al. reviewed the identification of the defect species based on ESR results [68]. They concluded that the g-factors for Ti^3+^ species and oxygen vacancies overlap.

At any rate, upon increasing the temperature of the calcination at the end of the sol–gel procedure, the intensity of this ESR signal decreased significantly, suggesting more ordered structures of the semiconductors. It appears that the magnitude of the ESR signal correlates to some extent with the magnitude of the weak absorption tail of the optical absorption spectra. Indeed, if one accepts that the narrow ESR signal around g ≈ 2.00 is connected to oxygen vacancies, it is plausible to assume that electronic transitions associated with these defects should give a relevant contribution to the weak absorption region, even if no distinct absorption band due to oxygen vacancies (around 500 nm/2.5 eV [66]) can be identified in the spectra of Figure 3 or Figure S4. It may be worth noting that the presence of reducing agents such as active carbon [32] or ammonia [69] was found to be responsible for the formation of oxygen vacancies during calcination of titania obtained from hydrolysis of titanium-isopropoxide. In our case, citric acid used in the gelling process could be also regarded as a reducing agent [70] that could behave analogously.

### 3.3. Characterization of the Fresh Pt-PtO_x_/TiO_2_ Samples

Pt contents of the fresh samples measured by ICP-OES varied between ±4% of the nominal value (0.7 weight%). In order to obtain information about the fresh semiconductor-co-catalyst systems TEM, XPS and ESR measurements were performed.

**Figure 5:**
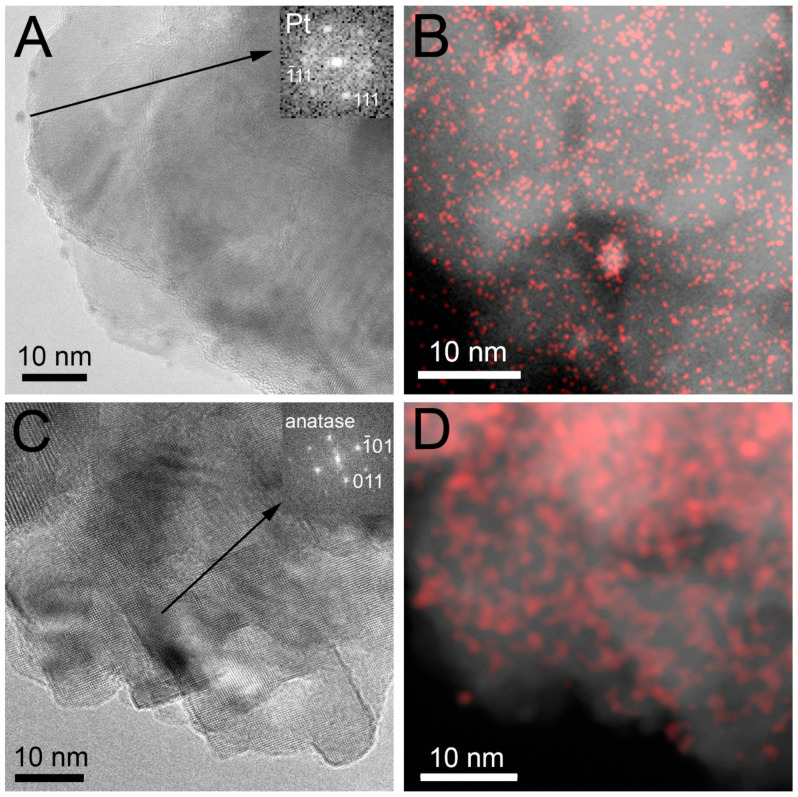
High-resolution scanning TEM (STEM) images and element maps of selected samples. (**A**) STEM image of sample SG500PtH2red. FFT (insert) shows <111> Pt reflections; (**B**) Pt element map of sample SG500PtH2red; (**C**) STEM image of sample SG500PtCalc. FFT (insert) shows <011> anatase reflections; (**D**) Pt element map of SG500PtCalc.

TEM studies indicated no observable change in the structure of the semiconductor upon Pt loading and activation. All the SG-based catalysts activated by hydrogen reduction (SG400PtH_2_red, SG450PtH_2_red and SG500PtH_2_red) were very similar to each other (see Figure S5A,C,E). A high-resolution scanning TEM (STEM) investigation of a selected sample (SG500PtH2red) demonstrated that the Pt particle size was 1–4 nm and the particles were evenly distributed on the surface (Figure 5A,B) in accordance with our previous work [60]. In particular, the Fourier transformation of the image of a particle on the surface of the support indeed revealed the <111> reflections of metallic Pt.

When the Pt co-catalyst was formed by calcination, Pt containing nanoparticles cannot be observed using conventional bright-field TEM [60] due to the little contrast difference between PtO*_x_* and TiO_2_ (also see Figure S5B,D,F). However, the presence of Pt-containing species could be identified using scanning TEM combined with Pt element mapping (Figure 5C,D). While no Pt- or PtO*_x_*-related feature was evident in the STEM images or their Fourier transforms, a network of relatively large Pt-containing patches was visualized on the element map, which suggests that the PtO*_x_* particles were amorphous and spread on the TiO_2_ surface. We note that all the samples with co-catalyst obtained by calcinations (SG400PtCalc, SG450PtCalc and SG500PtCalc) were also very similar to each other (see Figure S5B,D,F).

**Figure 6:**
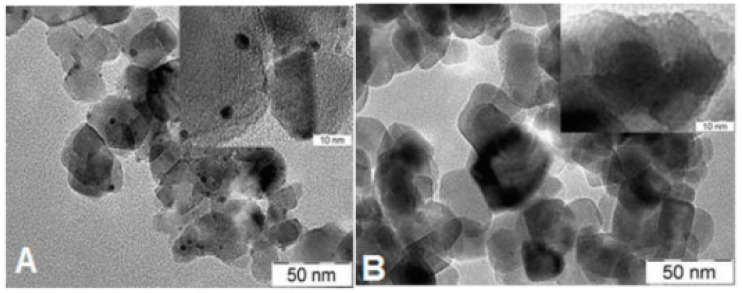
TEM images of P25-based Pt-PtO*_x_*/TiO_2_ samples. (**A**) P25PtH_2_red; (**B**) P25PtCalc.

TEM images of the fresh P25-based sample pair (i.e., co-catalyst formed by H_2_ reduction and calcination) are shown in Figure 6. As it was observed in the case of the SG supports, Pt particles are evident in the catalyst formed by reduction, while no Pt-related feature is identifiable on the calcined sample.

The apparent surface composition of the photocatalysts as well as the chemical states of the components was determined by XPS. The titanium content of all samples was exclusively in the Ti^4+^ ionic state (Ti 2p_3/2_ peak at 458.8 eV, assigned to TiO_2_) and no peak shape difference was evident between the SG or P25-based materials. The O1s spectrum mainly aroused from the TiO_2_ itself (the main O1s component was always found around 530 eV), although minor contributions from the hydroxyl groups on the TiO_2_ (O1s 531 eV), and to a lesser extent from the oxygen linked to carbon impurities (O1s 532.3–532.5 eV) were also detected. The carbon content of the samples accompanied by a tiny nitrogen content was attributed to contamination from exposure to the ambient.

Since the catalysts were exposed to ambient air after synthesis and activation by annealing in H_2_ or calcination, certain post-preparation oxidation of the Pt content is expected. Indeed, a gradual but slow reduction of the Pt content of the catalysts was observed during the XPS experiment, so for further discussion, we selected the spectra obtained after cca. 1 h X-ray exposure.

In order to evaluate the Pt 4f XPS spectra, first, a correction for the charge-transfer satellite of the Ti 3s peak (which is at 13.3 eV higher binding energy than the parent peak thus overlaps with the Pt 4f region) was applied. Then the spectra were modeled by a combination of a metallic contribution (a 4f_7/2_-4f_5/2_ spin-orbit doublet with an asymmetric line shape and a Pt 4f_7/2_ peak around or below 71 eV), a Pt^2+^ spin-orbit doublet (arising from PtO or Pt(OH)_2_ with the Pt 4f_7/2_ peak between 72–73 eV) and a Pt^4+^ doublet (from PtO_2_ with the Pt 4f_7/2_ peak at 74–75 eV) [44,71].

**Figure 7:**
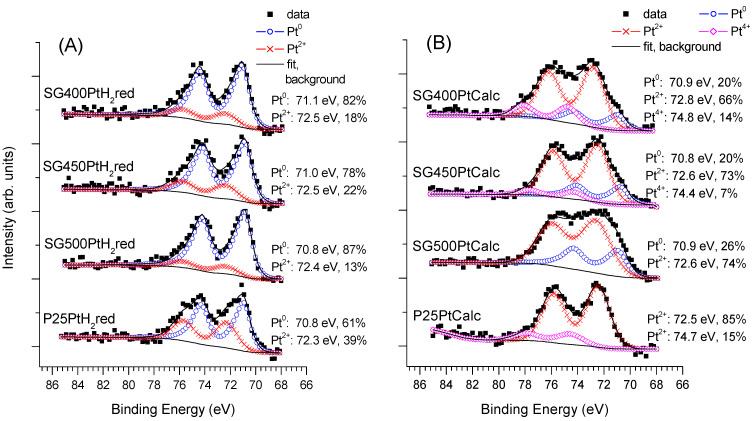
Pt 4f spectra of the fresh Pt-PtO*_x_*/TiO_2_ catalysts formed by (**A**): H_2_ reduction and (**B**): calcination. Spectra were recorded after cca. 1 h X-ray exposure.

The Pt 4f spectra for the sample series activated by H_2_ reduction are summarized in Figure 7A, while results for the series obtained by calcination are shown in Figure 7B. As far as the Pt content and its chemical states are concerned, the members of the similarly activated series of sol–gel-based samples were generally similar to each other, while clear deviations are evident for the P25-based catalysts.

In the case of the photocatalysts where the co-catalyst was formed by H_2_ reduction, metallic Pt gave the dominant chemical state, irrespectively to the support, accompanied by a certain amount of Pt^2+^. While the Pt^2+^ content of the catalysts on the sol–gel-based supports was relatively low, it was clearly higher in the case of the P25-supported samples. The same tendency is even more apparent if the Pt 4f spectra measured after minimal X-ray exposure are compared (see Supplementary Materials Figure S7A). Consequently, the P25 support has a clear stabilizing effect on the oxidized forms of Pt after reduction in H_2_ at 400 °C, or, in other words, Pt is more easily reducible on the SG supports than on P25. The observed dominance of the metallic phase of Pt is consistent with the results of the electron microscopic studies. Note that ionic Pt observed by XPS in minor quantities may not be observable by TEM.

When the PtO*_x_*/TiO_2_ samples were formed by calcination in air, Pt existed mainly in the form of Pt^2+^ in the fresh catalysts (Figure 7B) although a certain amount of Pt^4+^ and Pt^0^ could also be observed on the sol–gel-based supports according to the thermal disproportion of the Pt(NH_3_)_4_(NO_3_)_2_ precursor [72]. The reductive effect of the X-ray exposure was much more limited on the calcined catalysts (see Figure S7B in the Supplementary Materials for the spectra recorded after minimal X-ray exposure) than on their reduced counterparts: the main changes were a certain decrease of the Pt^4+^ content and a limited increase of the metallic contribution on the sol–gel supports. In the case of the P25-supported catalyst, no metallic Pt was detected even after 1 h of measurement time, confirming the enhanced stability of ionic Pt on the P25 material with respect to the sol–gel TiO_2_. These results are again in good agreement with the findings of TEM.

The most important results of quantitative analysis of the Pt content obtained from XPS data are summarized in Table 2.

The Pt content is expressed in weight% and the Pt/Ti ratio is also presented (Table 2). Data in Table 2 reveal that the apparent Pt content was in all investigated fresh samples considerably higher than the ICP-measured 0.7 weight%. It can be explained by the fine dispersion of the Pt species on the surface of the TiO_2_ particles. While the XPS-derived Pt content of the P25-supported reduced and calcined catalysts is very similar, suggesting a comparable dispersion of the Pt species, a notable difference is evident in the case of the fresh SG-based samples after the two activation treatments. The high apparent Pt content of the fresh calcined sol–gel-supported samples indicates the highly dispersed nature of the Pt in these systems. The composition data suggest that the Pt dispersion of the fresh calcined SG-supported catalysts increases with an increasing annealing temperature of the support.

The tendency observed for the apparent Pt content on these catalysts may point to a support calcination temperature-dependent change in the interaction of the Pt precursor with the SG support and its decomposition behavior upon co-catalyst activation by calcination. Both this feature and the enhanced stability of the ionic Pt species on the P25 material suggest that the surface chemistry of the sol–gel-based and the P25 supports is different.

**Figure 8:**
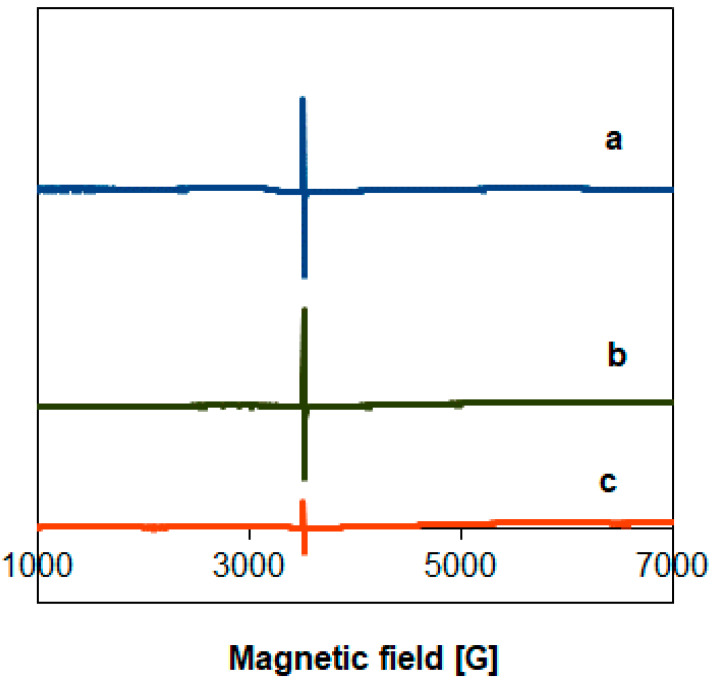
Effect of the co-catalyst forming by calcination in sol–gel-prepared samples on the ESR spectra. (**a**) SG400; (**b**) SG400PtCalc, (**c**) blank SG400 sample re-calcined at 400 °C.

The ESR response of the fresh Pt-PtO*_x_*/TiO_2_ catalysts was studied after co-catalyst formation by calcination or H_2_ reduction. Both the Pt-loaded P25 TiO_2_-based samples gave a very weak signal at the detection limit of the instrument, similar to the case of the bare supports depicted in Figure 4. ESR spectra of the sol–gel-based Pt-PtO*_x_*/TiO_2_ catalysts formed by either calcination or high-temperature H_2_ treatment showed the narrow line with a g value around 2.00. During the co-catalyst formation by calcination the ESR spectra of the parent SG TiO_2_ remained largely unchanged, as illustrated in Figure 8 for the case of the SG400-based systems (traces a and b). Interestingly, annealing of the parent TiO_2_ in the absence of the Pt precursor (blank sample re-calcined at 400 °C) resulted in some decrease of the signal (Figure 8, trace c).

As we found in our previous work, the high-temperature H_2_ treatment itself can produce this type of ESR signal even on sol–gel-prepared bare TiO_2_ [50], which observation is consistent with the general picture about the creation of oxygen vacancies [73]. Therefore, the presence of this narrow ESR peak in the case of the photocatalysts formed on sol–gel supports by H_2_ treatment (not shown) is not surprising. In fact, several authors in the literature assume that the oxygen vacancies responsible for the sharp ESR signal at g ≈ 2.00 may influence the surface redox properties of the TiO_2_ materials [74,75,76,77,78]. As such vacancies are detectable in the sol–gel supports even after preparation at 500 °C, but are missing from the P25 TiO_2_, their influence on the surface chemical properties can indeed explain some of the XPS observations, although microstructural differences as well as the always higher overall disorder of the sol–gel materials should also be considered.

### 3.4. Characterization of the Catalysts Recovered after the Reaction

**Figure 9:**
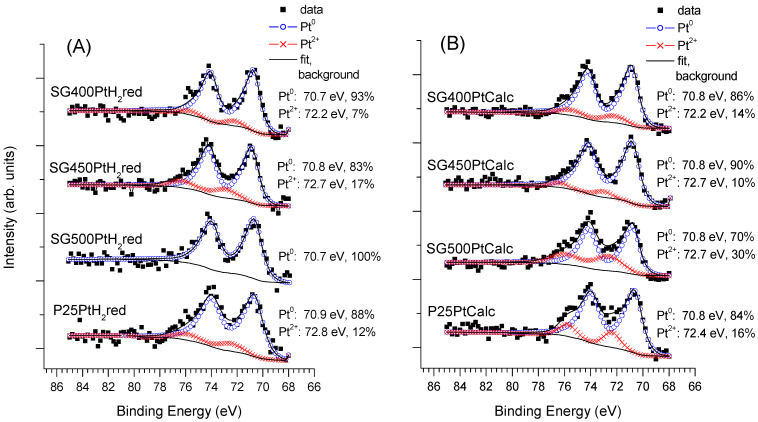
Pt 4f spectra of the recovered Pt-PtO*_x_*/TiO_2_ photocatalysts activated by (**A**) H_2_ reduction, (**B**): calcination. All spectra were collected after cca. 1 h X-ray exposure.

In Figure 9, Pt 4f spectra of the investigated photocatalysts recovered after the photocatalytic experiment are shown. The chemical state of the platinum content is rather similar in all recovered samples: All spectra are dominated by the metallic Pt signal, along with minor Pt^2+^ contributions. If the Pt co-catalyst was formed by H_2_ treatment, the Pt 4f spectra of the used sol–gel-based catalysts are very similar to those of the fresh ones (cf. Figure 7A and Figure 9A); the only difference is that the Pt content of the used samples is marginally more reduced than that of the fresh ones. A similar but more pronounced effect is evident for the P25-based sample.

On the contrary, a dramatic chemical state change occurred in the sample series activated by calcination (cf. Figure 7B and Figure 9B): instead of the ionic Pt forms characteristic for the fresh catalysts, metallic Pt dominated the spectra of the used catalysts, indicating that a very significant in situ reduction of platinum occurred during the photocatalytic reaction. This in situ reduction can be regarded as an analogue to the photodeposition process [43] or the metallization of ions preadsorbed on the semiconductor [79], although the reaction conditions, circumstances and mechanisms are clearly different. At any rate, the resulting Pt 4f spectra resemble closely those of the used H_2_ reduced photocatalysts. At the same time, Pt nanoparticles could be observed in TEM images of the recovered samples activated by calcination (Figure S6 in the Supplementary Materials), in contrast to the TEM images of fresh samples (Figure 5C and Figure 6B).

Comparison of data obtained by quantitative evaluation of the XPS spectra on the recovered samples and on the fresh ones (cf. columns “Fresh and “Recovered” in Table 2) reveals that the apparent Pt content always decreased during the photocatalytic reaction. In fact, for both the P25PtCalc and the SG400PtH_2_Red samples selected for analysis, ICP and XPS indicated similar Pt loss during the photocatalytic process, suggesting that dissolution and mechanical abrasion were the main reasons for Pt loss, while sintering of Pt (which also results in decreasing apparent Pt content) is less important. The quantitative analysis confirms the similarity of the used catalysts, regardless of whether they were initially formed by calcination or H_2_ treatment.

The ESR results on selected sol–gel-based PtO*_x_*/TiO_2_ samples before and after the photocatalytic reaction (which are presented in Figure S8 and shortly discussed in the Supplementary Materials, Section S3.3) also suggest that differences between catalysts activated by calcination or H_2_ treatment diminish under the reaction conditions.

## 4. Discussion

In Section 3.3, it was pointed out that the dispersion and the chemical state of Pt are rather similar on the catalysts formed by reduction, regardless of the type or the calcination temperature of the support. On the other hand, in catalysts activated by calcination, the Pt dispersion varies from values characteristic for the reduced catalysts (case of the PtP25Calc) to much higher values (case of the SG-based calcined systems). In addition, while the Pt content of the calcined catalysts is mainly ionic, the SG-based ones contain a certain amount of metallic Pt as well. This observation suggests that reduction of the Pt-PtO*_x_*/TiO_2_ system creates a generally similar interaction between the Pt species and the support, regardless of the support type or its thermal history, while co-catalyst activation by calcination emphasizes the surface chemical differences between the support materials.

In spite of the widely varying initial state of the studied photocatalysts (determined by the support and the activation treatment), samples recovered after the photocatalytic process are surprisingly similar both in terms of Pt dispersion, Pt chemical state or even the nature of the bulk defects, as described in Section 3.4. In the case of the catalysts formed by H_2_ treatment, the similarity of the fresh and the used samples indicates that the predominantly metallic state of Pt, probably along with some Pt^2+^ species, represents the active state of the catalysts under the reaction conditions. The drastic and almost complete transformation of the Pt content of the catalysts activated by calcination towards the same active state during the reaction conditions demonstrates the universal nature of the in situ photoreduction. We believe therefore that the essentially identical catalytic performance of the sol–gel TiO_2_-based photocatalysts formed by reduction or calcination is the result of an in situ transformation towards a general active surface state. The somewhat lower hydrogen generation rate of the PtP25Calc catalyst with respect to its H_2_ treated counterpart is probably connected to the higher ionic Pt content of the former, which may be the result of the stabilization effect of the P25 material on oxidized Pt species, as observed in Section 3.3.

Nevertheless, the fact that the gradual decrease of disorder of the SG supports upon increasing preparation temperature (as indicated by the UV-Vis absorption data (see Figure 3 and the related description)) is not reflected by changes in the catalytic activity, still requires explanation. The results discussed so far indicate that there is no correlation between the nature of the Pt content of the catalyst in its active state and the disorder of the support. Thus, it cannot be stated that differences in the dispersion or chemical state of the Pt content counterbalance the effect of the improving structural quality of the support. Instead, we believe that there is a main structural feature in the sol–gel-based material which is not really improved by the increasing preparation temperature (at least in the 400–500 °C range) and which limits its photocatalytic performance. We assume that this distinctive feature is the partially coalesced multi-grained structure of the SG supports, which necessarily contains numerous internal grain boundary regions with lots of localized electronic states, which certainly promote trapping and recombination of the photogenerated charge carriers. Furthermore, the strong and narrow ESR signal around g ≈ 2.00 characteristic for the SG supports may contain contributions from these internal boundary regions, which are difficult to eliminate even by repeated calcination treatments (see Figure 8). This is in contrast with the P25 material, in which these internal boundaries are largely missing. Indeed, P25 which is regarded as “the standard photocatalyst” due to its relatively high activity in a multitude of reactions [62] is a flame-synthesized TiO_2_ containing anatase, rutile and some amorphous material containing a very low concentration of small size bulk defects [80].

Nevertheless, the decrease of the specific surface area of the SG500-based samples with respect to the other support materials (Table 1) may also influence the performance of the SG500-based photocatalysts. Namely, increasing the crystalline quality of the semiconductor by high-temperature treatments can be counterbalanced by the subsequent loss of the surface area. In this sense the order of activity normalized to the specific surface (H_2_,_SG500_ > H_2,SG450_ ≅ H_2,SG400_) would certainly reflect improved semiconductor structure of the SG500 TiO_2_, even if the above discussion about the role of the disordered internal boundary regions still remains valid.

As our catalysts obtained from sol–gel-based TiO_2_ were less active than those obtained from P25, we believe that the advantages of the sol–gel method can be exploited more in the production of TiO_2_ materials doped with different elements or TiO_2_-containing composites than in the production of pure TiO_2_.

## 5. Conclusions

In this work, a series of TiO_2_ photocatalyst supports were prepared by a sol–gel-based method and subsequent annealing at 400, 450 and 500 °C in air. In order to form the photocatalyst, Pt co-catalyst was loaded by impregnation and activated by either H_2_ reduction at 400 °C or calcination at 300 °C. Structural, compositional as well as catalytic features of these catalysts were compared to those measured on similarly activated but flame-synthesized P25 TiO_2_-supported counterparts.

The sol–gel synthesis followed by calcination resulted in TiO_2_ supports containing exclusively the anatase phase, while in the case of the P25 TiO_2_ the expected rutile-anatase mixture was observed. Optical absorption measurements indicated significant disorder in the sol–gel-based material calcined at 400 °C. ESR investigations suggested that oxygen vacancies occupied by single electrons were important contributors to the overall disorder. Nevertheless, increasing the calcination temperature in the 400–500 °C range drastically decreased the extent of the disorder and eliminated the ESR-active oxygen vacancies, while the P25 TiO_2_ was found to be even more ordered.

Electron microscopy and XPS analysis of the fresh catalysts revealed that Pt was predominantly metallic on all catalysts formed by reduction and was mainly ionic on the ones activated by calcination. Nevertheless, dispersion differences of ionic Pt on the sol–gel and the P25 support, as well as the enhanced ability of the latter to stabilize ionic Pt, indicated a difference between the surface chemical properties of the support materials.

The activity of the Pt-containing homemade sol–gel-based TiO_2_ samples remained always lower than that obtained on the P25-supported photocatalysts, even if the H_2_ evolution rate values were in the same order of magnitude for the two support materials. More surprisingly, essentially no difference was found between the H_2_ production over the SG-based catalysts formed by calcination or hydrogen treatment. XPS and ESR investigations of the used SG-based catalysts indicated that the dispersion and the chemical state of Pt, as well as the nature of the oxygen vacancies, became rather similar for the calcined and the reduced samples during the photocatalytic reaction, which explained their similar photocatalytic performance. The inferior catalytic performance of the sol–gel TiO_2_-based samples with respect to their P25-based counterparts was therefore attributed to a structural feature of the SG-supports, which could not be significantly improved by the increasing preparation temperature (at least in the range of 400–500 °C) and which limited the catalytic activity even if the extent of the structural disorder was decreased. We identified the partially coalesced multi-grained structure of the sol–gel-based supports as this distinctive feature, containing numerous internal grain boundary regions with lots of charge carrier trapping localized states. A further factor interfering with the catalytic performance of the sol–gel-based systems was the decrease of their specific surface area with the increasing calcination temperature.

## Figures and Tables

**Table 1:** Textural and structural properties of the studied TiO_2_ support materials.

Type of TiO_2_	SSA, m^2^/g	Crystalline Phases ^b^, %		Grain Size, nm	
		Anatase	Rutile	Anatase	Rutile
SG400	45 ± 10 ^a^	100	-	15–20 ^c^	-
SG450	47 ± 10 ^a^	100	-	15–30 ^c^	-
SG500	28 ± 10 ^a^	100	-	20–30 ^c^	-
P25	50 ± 10	82	18	24 ^b^	45 ^b^

^a^ average of two parallel preparations. ^b^ calculated from XRD measurements. ^c^ based on TEM images (see in Figure S3 in Supplementary Materials).

**Table 2:** Quantitative analysis of the Pt content obtained from X-ray photoelectron spectroscopy (XPS) data of the fresh and recovered catalyst samples.

Catalyst	Fresh		Recovered	
	Pt Content (Weight%)	Pt/Ti Ratio	Pt Content (Weight%)	Pt/Ti Ratio
SG400PtH_2_red	2.2	1:95 = 0.011	1.5	1:150 = 0.007
SG400PtCalc	3.0	1:74 = 0.014	1.9	1:117 = 0.009
SG450PtH_2_red	2.1	1:104 = 0.010	1.5	1:146 = 0.007
SG450PtCalc	3.6	1:62 = 0.016	2.0	1:108 = 0.009
SG500PtH_2_red	2.4	1:89 = 0.011	1.5	1:140 = 0.007
SG500PtCalc	4.2	1:50 = 0.020	1.9	1:115 = 0.009
P25PtH_2_red	2.0	1:120 = 0.008	1.5	1:139 = 0.007
P25PtCalc	2.2	1:102 = 0.010	1.8	1:150 = 0.007

## Supplementary Materials

The following are available online at https://www.mdpi.com/1996-1944/14/4/943/s1, Figure S1: Scheme of the photocatalytic reactor system. The LED light illuminated part of the reactor system (reactor on the left side) was not used in the present experiments, Figure S2: Raman spectra of the bare TiO_2_ samples. a: P25; b: sol–gel precipitation followed by calcination at 400 °C (SG400); c: sol–gel precipitation followed by calcination at 450 °C (SG500), d: sol–gel precipitation followed by calcination at 500 °C (SG500), Figure S3: TEM images of TiO_2_ samples prepared by sol–gel precipitation followed by calcination. (**A**) calcined at 400 °C (SG400); (**B**) calcined at 450 °C (SG450); (**C**) calcined at 500 °C (SG500); (**D**) P25, Figure S4: Analysis of the diffuse reflectance spectra of the SG400, SG450, SG500 and the P25 materials. (**A**): Tauc plot representation showing the fundamental optical band gaps. For clarity, the curves are vertically shifted with respect to each other. (**B**): logarithmic plots of the Kubelka–Munk functions; red lines: fits the linear segments. Red numbers at the linear segments: reverse of the slope (Urbach energy). For clarity, the curves are vertically shifted with respect to each other. Insert: logarithmic plots of the Kubelka–Munk functions normalized to the maximum around 4 eV, Figure S5: TEM images of fresh sol–gel based Pt-PtO*_x_*/TiO_2_ samples. (**A**) SG400PtH_2_red; (**B**) SG400PtCalc; (**C**) SG450PtH_2_red; (**D**) SG450PtCalc; (**E**) SG500PtH_2_red; (**F**) SG500PtCalc, Figure S6: TEM images of the recovered PtO*_x_*/TiO_2_ catalysts. TiO_2_: P25. (**A**) co-catalyst formed by H_2_ reduction. (**B**) co-catalyst formed by calcination in air, Figure S7: Pt 4f spectra of the fresh Pt-PtO*_x_*/TiO_2_ catalysts formed by (**A**) H_2_ reduction and (**B**) calcination. Spectra were recorded after minimal X-ray exposure, Figure S8: Comparison of electron spin resonance (ESR) results of selected sol–gel based fresh and recovered Pt-PtO*_x_*/TiO_2_ catalysts. A: line width; B: g values of samples from co-catalyst formation by calcination; C: g values of samples from co-catalyst formation by high-temperature H_2_ treatment, Table S1: Results of simulations.
