# Supplementary material for: Effect of the Microstructure of the Semiconductor Support on the Photocatalytic Performance of the Pt-PtOx/TiO2 Catalyst System
Source: Materials (Basel). 2021 Feb 17;14(4):943. doi: 10.3390/ma14040943 (PMC7921961; doi:10.3390/ma14040943)
Supplement: Supplementary file 1 [file materials-14-00943-s001.pdf]

materials

MDPI

Supplementary Materials

Effect of the Microstructure of the Semiconductor Support on the Photocatalytic Performance of the Pt-PtO$_x$/TiO$_2$ Catalyst System

Katalin Majrik [1], Zoltán Pászti [1,*], László Korecz [1], Judith Mihály [1], Zoltán May [1], Péter Németh [1,2], Catia Cannilla [3], Giuseppe Bonura [3], Francesco Frusteri [3], András Tompos [1] and Emília Tálas [1]

[1] Research Centre for Natural Sciences, Institute of Materials and Environmental Chemistry, Eötvös Loránd Research Network (ELKH) H-1117 Budapest, Magyar tudósok körútja 2, Hungary; majrik.katalin@ttk.hu (K.M.); korecz.laszlo@ttk.hu (L.K.); mihaly.judith@ttk.hu (J.M.); may.zoltan@ttk.hu (Z.M.); nemeth.peter@ttk.hu (P.N.); tompos.andras@ttk.mta.hu (A.T.); talas.emilia@ttk.hu (E.T.)

[2] Department of Earth and Environmental Sciences, University of Pannonia, H-8200 Veszprém, Egyetem út 10, Hungary

[3] National Council of Research–CNR-ITAE, ''Nicola Giordano'', Via S. Lucia 5, 98126 Messina, Italy; cannilla@itae.cnr.it (C.C.); giuseppe.bonura@itae.cnr.it (G.B.); francesco.frusteri@cnr.it (F.F.)

* Correspondence: paszti.zoltan@ttk.hu; Tel.: +36 1 382 6412

S1. Experimental

Raman spectra were recorded with a dynamically aligned Bio-Rad (Digilab) dedicated FT-Raman spectrometer equipped with a Spectra-Physics Nd-YAG-laser (1064 nm) and a high sensitivity liquid-N$_2$ cooled Ge detector. The laser power used was about 250 mW in the samples. The resolution of the Raman instrument was ca. 4 cm^{-1} and a backscattered geometry was used. For each spectrum, 256 individual spectra were averaged.

A Philips CM12 instrument equipped with a high-resolution camera was used to acquire and elaborate the TEM images. Powdered samples were dispersed in 2-propanol under ultrasound irradiation and the resulting suspension dropwise was deposited on a holey carbon-coated support grid.

The ESR experiments were performed with a Bruker Elexsys E500 X-band spectrometer. A typical microwave power of 1 mW and 1 G magnetic field modulation at ambient temperature were used. The magnetic field was calibrated with an NMR field meter. Signal intensity, linewidth and g-factor (spectroscopic splitting factor) values were used to characterize the samples. The knowledge of the g-factor can give information about a paramagnetic center's electronic structure.

X-ray photoelectron spectroscopy (XPS) measurements were carried out using an EA125 electron spectrometer manufactured by OMICRON Nanotechnology GmbH (Germany). The photoelectrons were excited by both MgKα (1253.6 eV) and AlKα (1486.6 eV) radiation. Spectra were recorded in the Constant Analyzer Energy mode of the energy analyser with 30 eV pass energy resulting in a spectral resolution around 1 eV. For XPS experiments the samples in the form of fine powder were suspended in isopropanol. Drops of this suspension were placed on standard OMICRON sample plates; after evaporation of the solvent catalyst coatings with sufficient adhesion and electric conductivity were obtained. Effects of possible electric charging were compensated by adjusting the binding energy of the main component of the C 1s envelope (hydrocarbons) to 285.0 eV. By this choice both the Ti 2p$_{3/2}$ and the O 1s binding energies coincided with the range expected for TiO$_2$, confirming the reliability of the calibration. Chemical states of the elements were deduced from high-resolution

spectra using XPS databases [1,2]. Quantification was performed using a combination of CasaXPS [3] and XPSMultiQuant [4,5].

The photocatalytic reaction in the UV-visible region was carried out in a cylindrical quartz reactor (140 mm in height and 60 mm in diameter) equipped with magnetic stirrers, gas inputs and outputs. Osram HQL de luxe 125W lamps were used as light sources operated. Nitrogen gas with 20 cm^3/min flow rate was continuously bubbled through the reactor. The initial concentration of methanol was 6 v/v% in distilled water. The reaction was carried out at room temperature. The amount of catalyst and the reaction volume was 0.140 g and 260 cm^3, respectively. For experiments in the visible light region, a homemade LED light source operating at four distinct wavelengths (400, 420, 440, 450 nm) is available but this part of the reactor system was not used in the present experiments. In this latter part of the equipment, the LED light source is immersed in a UV-Consultig Peschl UV-Reactor System 1 photoreactor equipped with gas inputs and outputs. The reaction setup is depicted in Figure S1. GC (Agilent 7820A) equipped with SUPELCO Carboxen 1010 column and TCD detector was used to follow the H_2 production. The internal standard of the GC analysis was argon gas added to the vapor–gas mixture before the GC sampling valve. The H_2 production was expressed as H_2 production rate (mmol h^{-1}). The reaction was monitored for 270 min. After the photocatalytic reaction, the samples were recovered from the aqueous methanol solution by centrifuging, washing with 3 × 50 cm^3 absolute ethanol followed by drying under N_2 flow. Then the recovered samples were also characterized by ESR, XPS and/or TEM techniques.

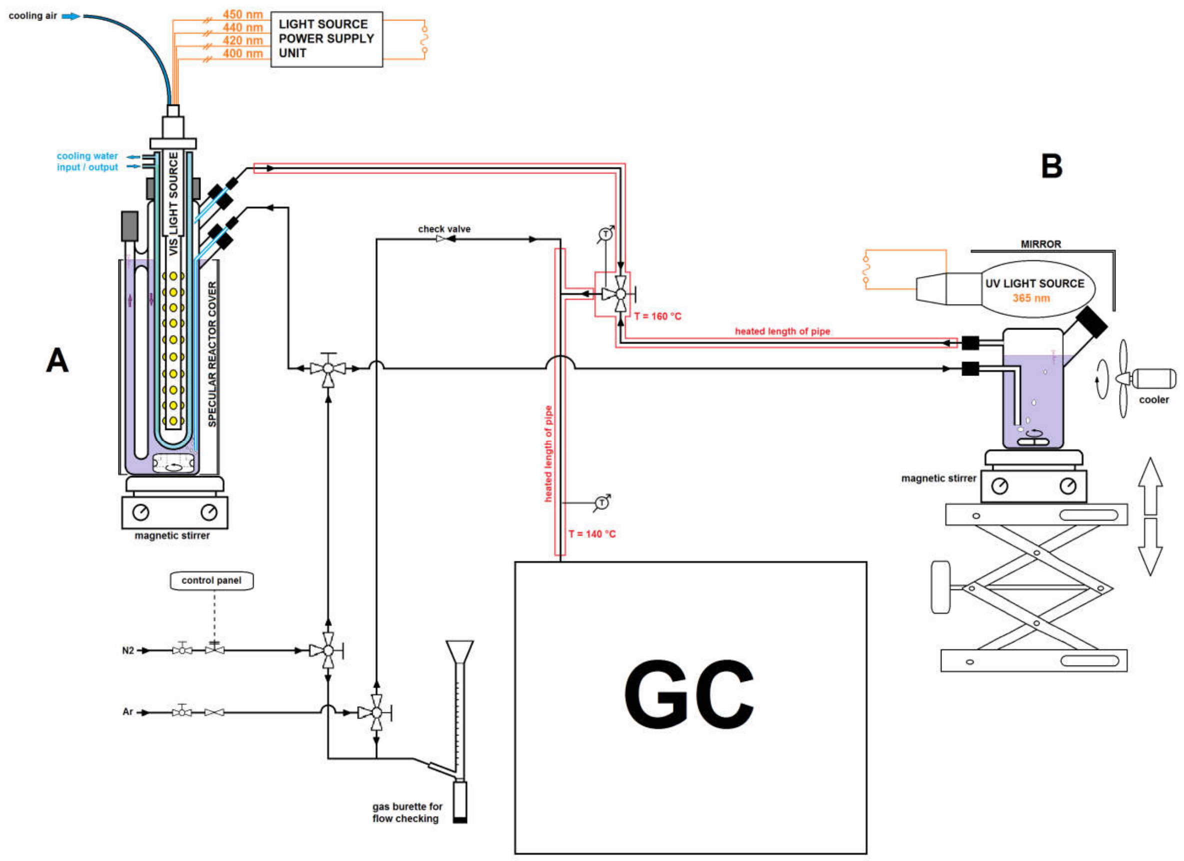

Figure S1. Scheme of the photocatalytic reactor system. The LED light illuminated part of the reactor system (reactor on the left side) was not used in the present experiments.

S2. Characterization of Bare TiO_2

S2.1 Raman Spectroscopic Measurements

The results of Raman spectroscopic measurements (Figure S2) are consistent with the XRD results (Table 1 in the main text); our sol–gel prepared TiO_2 samples contained exclusively anatase as a crystalline phase while P25 contained anatase and rutile phases.

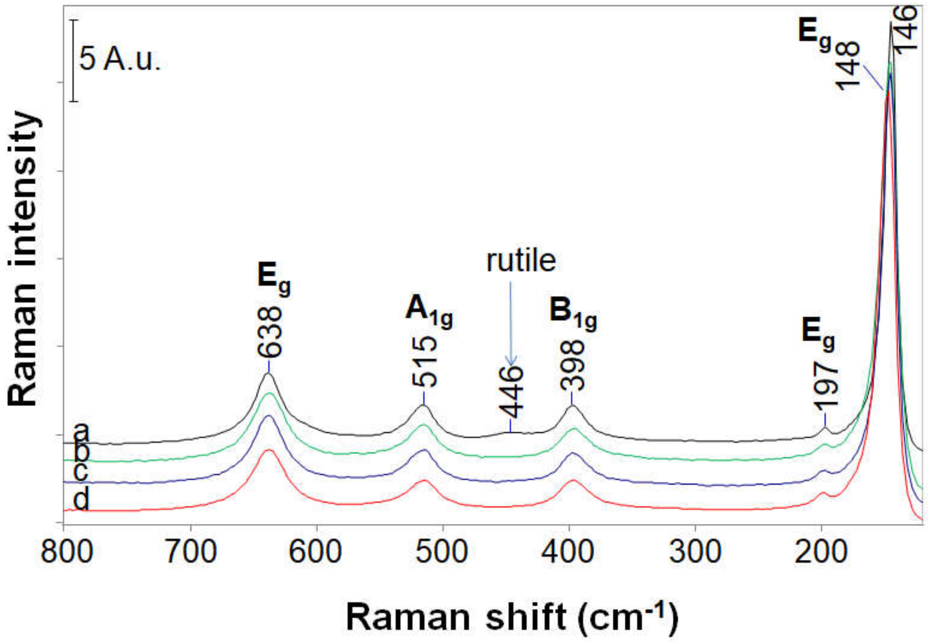

Figure S2. Raman spectra of the bare TiO_2 samples. a: P25; b: sol–gel precipitation followed by calcination at 400 °C (SG400); c: sol–gel precipitation followed by calcination at 450 °C (SG500), d: sol–gel precipitation followed by calcination at 500 °C (SG500).

S2.2 TEM Measurements

Figure S3 shows the TEM images of TiO_2 samples prepared by sol–gel precipitation followed by calcination. The polycrystalline anatase structure with a particle size of 10–40 nm was obtained.

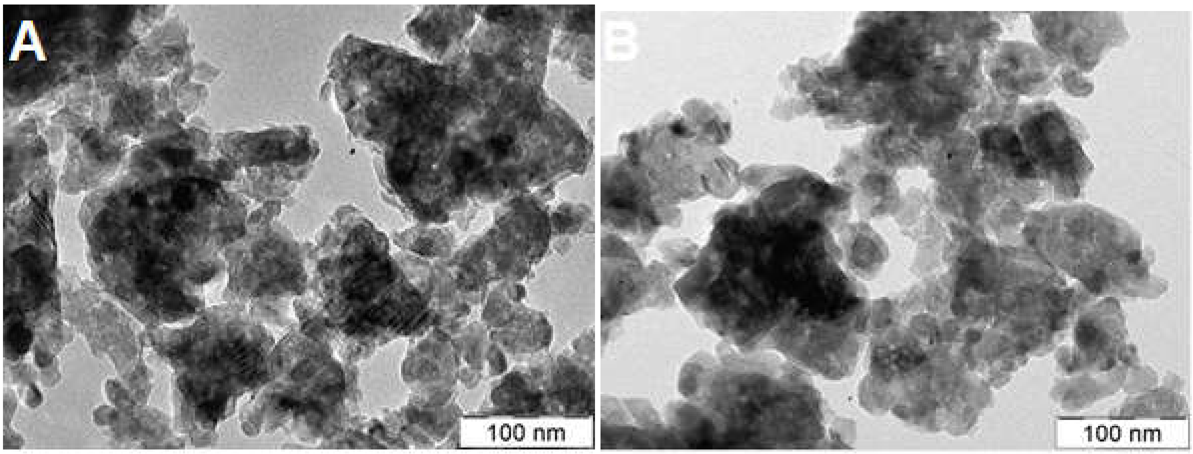

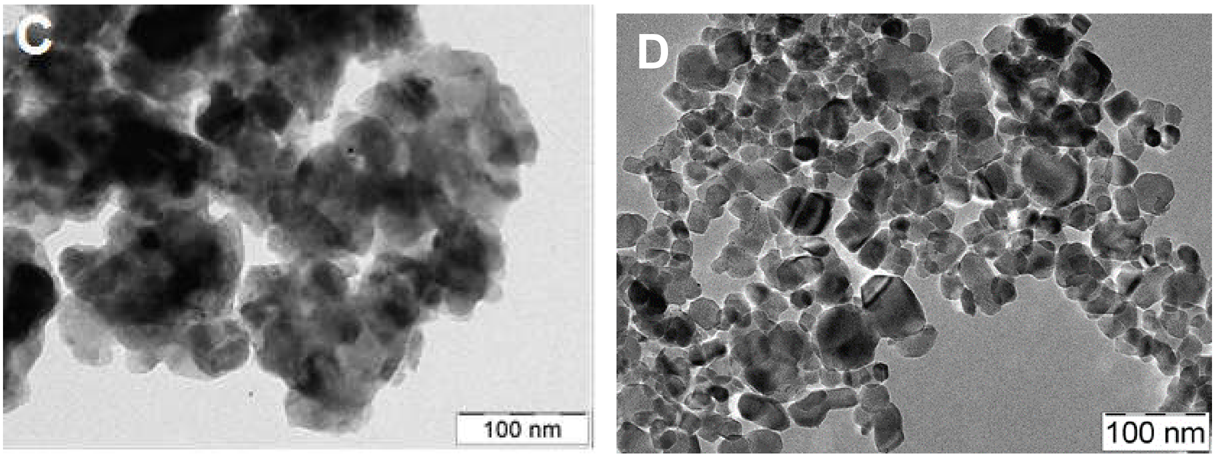

Figure S3. TEM images of TiO_2 samples prepared by sol–gel precipitation followed by calcination. (**A**) calcined at 400 °C (SG400); (**B**) calcined at 450 °C (SG450); (**C**) calcined at 500 °C (SG500); (**D**) P25.

S2.3. Evaluation of the Optical Absorption Spectra

In order to estimate the optical band gaps of the four TiO_2 materials, the standard procedure of processing the diffuse reflectance spectra [6] was followed.

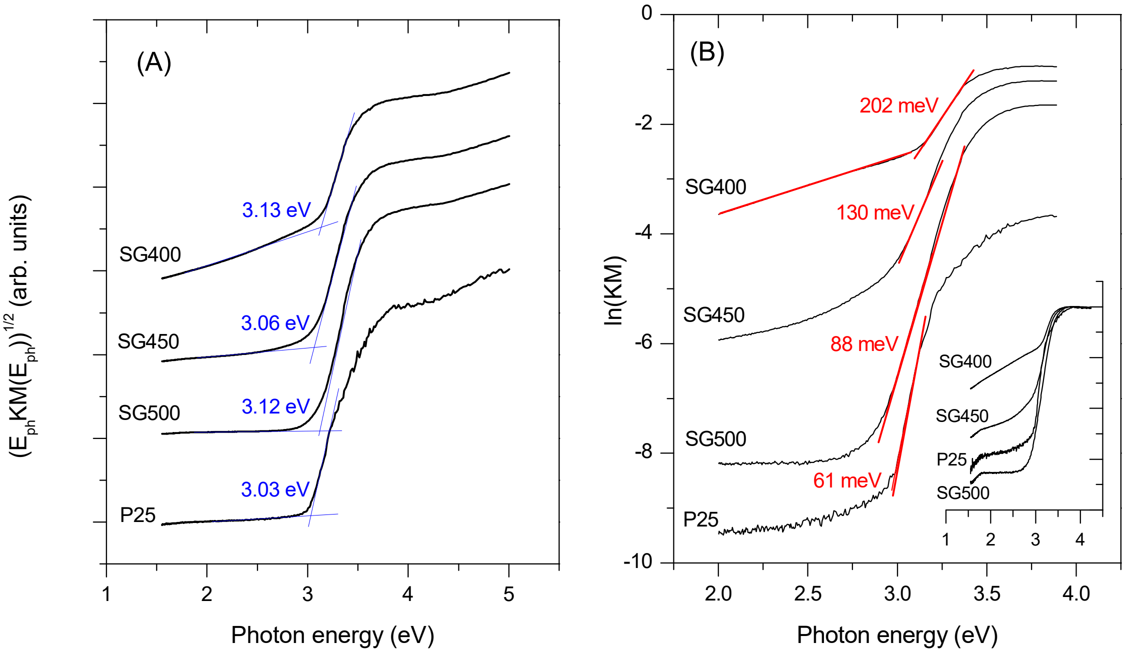

Figure S4. Analysis of the diffuse reflectance spectra of the SG400, SG450, SG500 and the P25 materials. (**A**): Tauc plot representation showing the fundamental optical band gaps. For clarity, the curves are vertically shifted with respect to each other. (**B**): logarithmic plots of the Kubelka–Munk functions; red lines: fits the linear segments. Red numbers at the linear segments: reverse of the slope (Urbach energy). For clarity, the curves are vertically shifted with respect to each other. Insert: logarithmic plots of the Kubelka–Munk functions normalized to the maximum around 4 eV.

First, reflectance data were transformed into the Kubelka–Munk function ($KM(R) = (1-R)^2/2R$, where R is the measured reflectance), which represents the wavelength dependence of the absorption coefficient. As the major crystalline phase in all four materials is anatase with indirect bandgap [7], the quantity $(KM(R)E_{photon})^{1/2}$ was plotted as the function of the photon energy (E_{photon}) and the intersection of its linear fit in the absorption edge region with the baseline was determined (Tauc plot method

[8]). Note that in the case of electronic transitions at interfaces, where momentum non-conservation can occur [9] or in amorphous semiconductors [8], the same formula describes the absorption edge. The Tauc plots are shown in Figure S4A. The resulting band gap values are very close to each other and are around 3.00–3.15 eV.

According to the theory of disordered semiconductors [8], defects (*i.e.* deviations from the normal coordination in terms of positions and chemical identity of atoms) inevitably generate localized electronic states near the band edges of the ideal material, which – in terms of the density of states – form more or less exponential tails into the bandgap. In addition, deep localized states also occur in the bandgap region. Optical excitations involving these localized states are necessarily observable in the absorption spectrum. As a result, the absorption spectrum of disordered semiconductors contains three distinct regions: (i) at photon energies much lower than the bandgap transitions between the tail states give a weak absorption tail which is sometimes exponential; (ii) around the bandgap energy, another exponential segment exists due to transitions involving tail and non-localized band states which are known as the Urbach tail; and (iii) above the bandgap, energy absorption is due to interband transitions and the spectrum follows the Tauc rule. The magnitude of the absorption in the region (i) may be regarded as a tentative indicator of the extent of disorder in the material.

The logarithmic representation of the Kubelka–Munk functions of the materials studied in this work is shown in Figure S4B. It is obvious either from the absorbance data (Figure 3 of the main paper) or the semi-logarithmic plots of the Kubelka–Munk functions normalized to each other (insert of Figure S3B) that absorption in the weak absorption region (i.e., well below 3 eV) is the weakest for the SG500 material, only slightly higher for P25, considerably higher for SG450 and the highest for SG400. This means that while the SG500 and the P25 samples are relatively free from defect-induced deep localized states, the densities of state tails penetrating deeply into the bandgap are prominent features of the electronic structure of SG400, and SG450 represents an intermediate situation. In all materials, a pronounced linear part can be identified in the logarithmic representation of the Kubelka–Munk function near the bandgap energy. This exponential tail of the absorption edge, also known as the Urbach tail, is a consequence of the disorder-induced smearing of the band edges. The slope of the linear segment of the semi-logarithmic plot of the absorption edge is a direct measure of the extent of the disorder [10] or the width of the disorder-induced tails of the density of states [8]: the lower the slope (or the higher its reverse, the Urbach width or Urbach energy), the higher is the disorder in the system. Indeed, the Urbach energy is lowest for the P25 sample, slightly higher for the SG500 and significantly increases through SG450 to SG400. This order again confirms that disorder-related localized electronic states dominate the optical response of the SG400 material and even in SG500 their role is more prevalent than in P25.

It must be noted that even if these disorder-induced electronic states may offer optical absorption well into the visible range, the generated electron-hole pairs remain localized either at or near the surface of the semiconductor or even in its bulk and their chance for participation in a photocatalytic process remains very low. On the other hand, these states can act as electron traps and/or recombination centers, which further diminishes the photocatalytic performance of the material.

S3. Characterization of the Platinum Containing Samples

S3.1 TEM Measurements

Figure S5 shows the TEM images of the fresh sol–gel based Pt-PtO_x/TiO_2 samples. According to Figure S5, TiO_2 was consistent with polycrystalline anatase having a particle size of 10–40 nm.

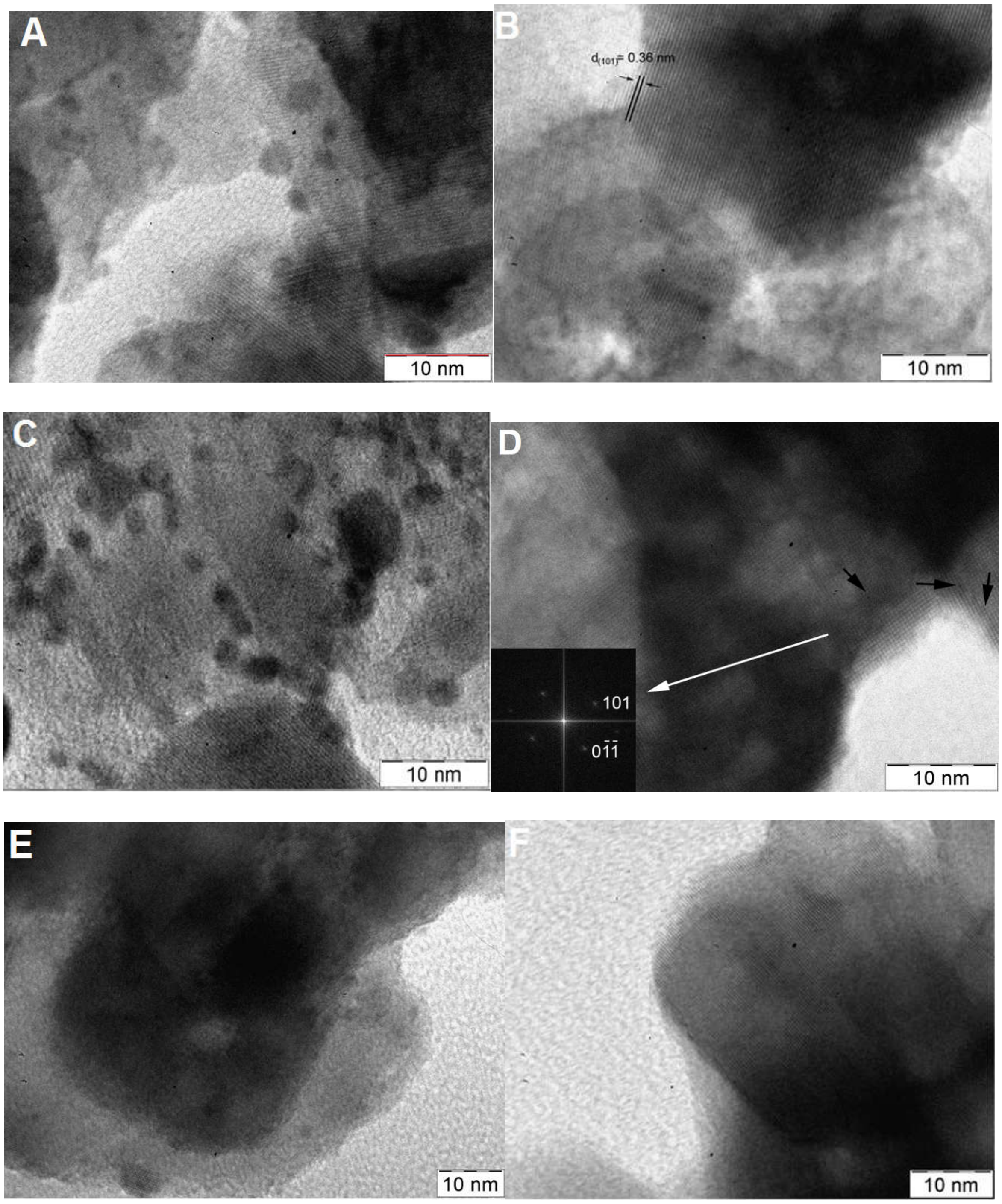

Figure S5. TEM images of fresh sol–gel based Pt-PtO$_x$/TiO$_2$ samples. (**A**) SG400PtH$_2$red; (**B**) SG400PtCalc; (**C**) SG450PtH$_2$red; (**D**) SG450PtCalc; (**E**) SG500PtH$_2$red; (**F**) SG500PtCalc.

All the samples (of SG400PtH$_2$red, SG450PtH$_2$red and SG500PtH$_2$red) with co-catalyst obtained by hydrogen reduction were very similar to each other (see Figure S5A, C, E) with a few features in the 2–4 nm range giving darker contrast, which were identified as reduced Pt particles, whose presence was also confirmed by XPS. When the Pt was formed by calcination, Pt containing nanoparticles cannot be observed using conventional bright-field TEM [11] due to the little contrast difference between PtO$_x$ and TiO$_2$ (also see Figure S5B, D, F).

According to the TEM images of recovered samples, Pt-like nanoparticles can be observed even in the case of PtO$_x$/TiO$_2$ sample formed by calcination in air (Figure S6).

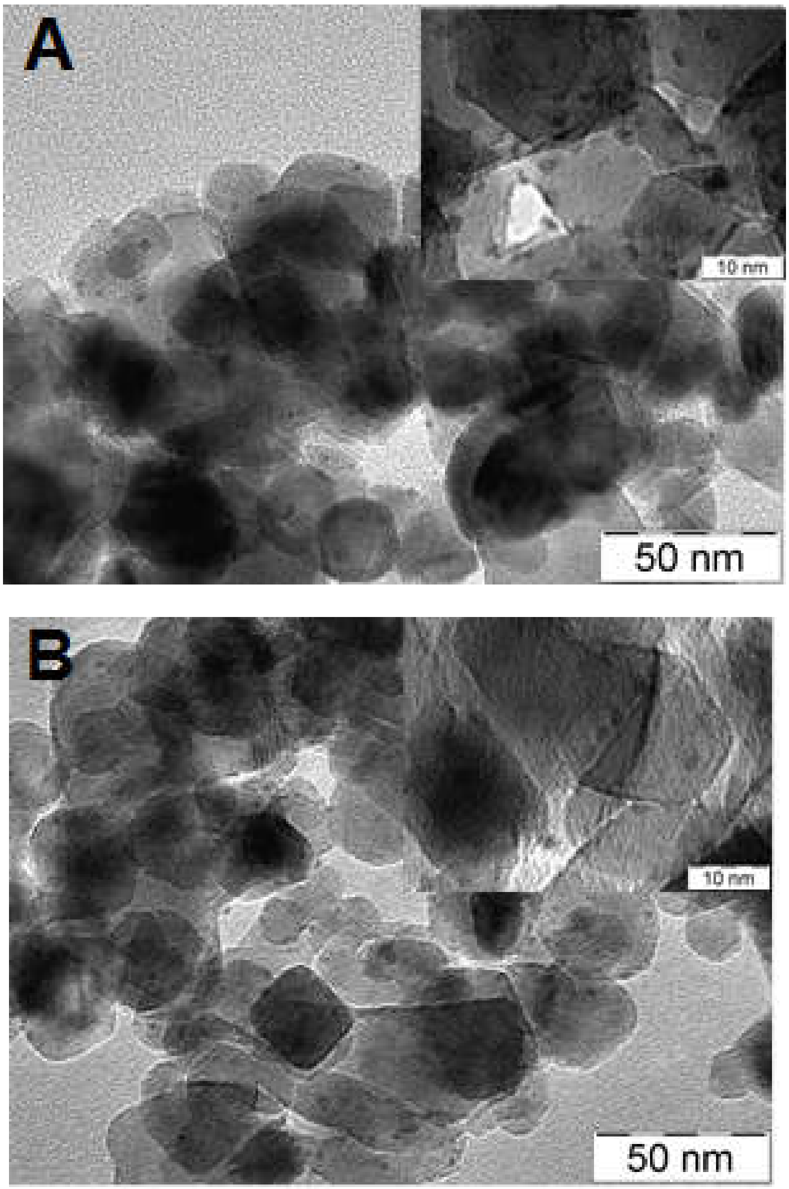

Figure S6. TEM images of the recovered Pt-PtO_x/TiO_2 catalysts. TiO_2: P25. (**A**) co-catalyst formed by H_2 reduction. (**B**) co-catalyst formed by calcination in air.

S3.2 XPS Measurements

While the Pt 4f core-level spectra of the Pt-loaded TiO_2 samples recorded after 1h X-ray exposure are compared in Figure 7 of the main text, Figure S7 shows spectra obtained immediately after the beginning of the XPS measurement, so the in-radiation reduction of the Pt content is minimal.

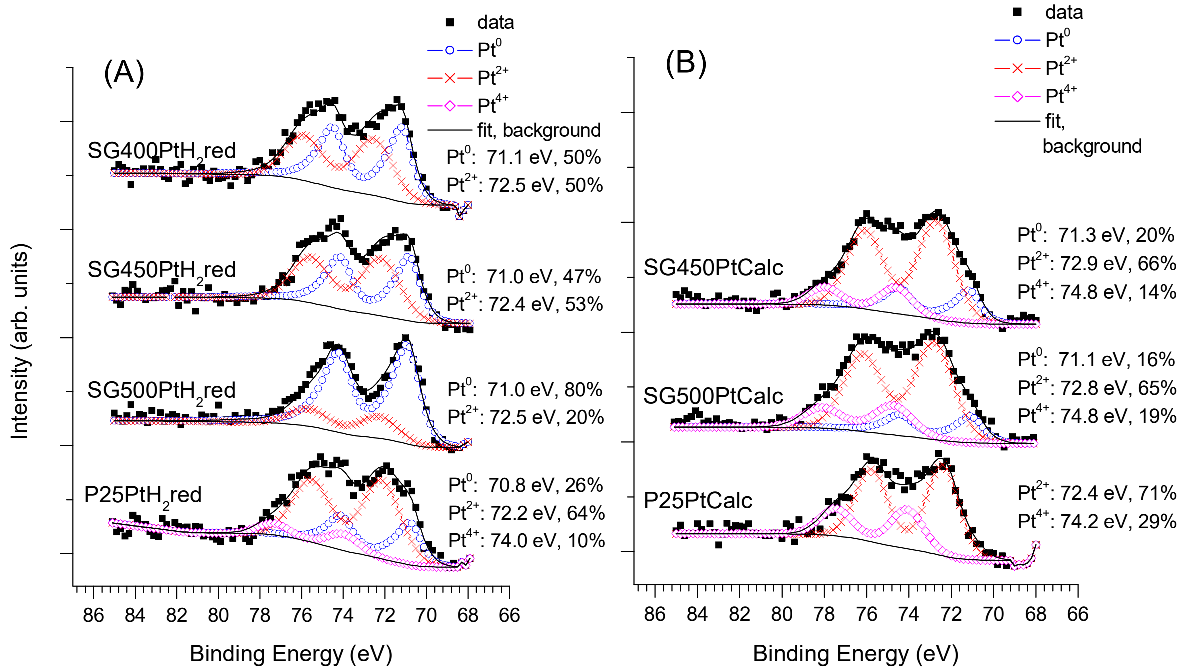

Figure S7. Pt 4f spectra of the fresh Pt-PtO_x/TiO_2 catalysts formed by (**A**) H_2 reduction and (**B**) calcination. Spectra were recorded after minimal X-ray exposure.

Upon comparison of Figures 7A of the main text and S7A of the Supplementary Materials, it is evident that in the case of the catalysts prepared by reduction, the Pt content is very easily reducible even under the UHV conditions of the XPS experiment (probably due to the slight heating effect of the X-ray source). Nevertheless, while in the case of the SG400 and SG450-supported reduced samples, a significant decrease of the Pt^{2+} contribution is evident during the measurement, the SG500-supported reduced sample is more reduced initially and the further reduction remains limited. This indicates that in the Pt-loaded SG400 or SG450 catalysts, either the initial reduction was less complete (which is not very probable, as when a Pt-oxide fraction is not reduced at 400 °C in hydrogen, it should be stable upon the more gentle conditions of the XPS experiment) or the supports prepared at lower temperatures facilitate the reoxidation of Pt upon air exposure to a higher extent compared to the SG500 material. On the other hand, the PtP25 sample formed by reduction was indeed quite oxidized at the beginning of the XPS experiment and was reduced under the measurement conditions to a much smaller extent than its SG-based counterparts, which suggests that the P25 material has a more pronounced stabilization effect on the ionic Pt species than the SG-based materials.

While in general, the spectral changes of the Pt-loaded SG samples formed by calcination during the XPS experiment remained much more limited (see Figure 7B of the main text and Figure S7B of the Supplementary Materials), it appears that not only the Pt^{2+} but even the Pt^{4+} state is more stable on the SG400 or SG450 supports than on the SG500. In the case of the P25-based catalyst activated by calcination, only a certain decrease of the Pt^{4+} content is evident during the XPS measurement and the metallic Pt, which was detected on the calcined SG-based catalysts even at the very beginning of the XPS experiment, and does not appear even after long measurement times.

S3.3 ESR Measurements

As the line shape measured for the Pt-loaded photocatalyst samples was not perfect Lorentzian, we carried out a series of simulations to find the best fit, the quality of the simulation was characterized by R (coefficient of correlation). We performed fits with isotropic (Lorentzian, Gaussian, superposition of Lorentzian and Gaussian, dispersion

distorted Lorentzian) and rhombic (Lorentzian, Gaussian) models. The unresolved rhombic symmetry model gives the best results (see Table S1).

Table S1. Results of simulations.

SG400PtCalc			SG400PtH$_2$red			SG450PtCalc			SG450PtH$_2$red	
	R			R			R			R
Lorentz	0.9872		Lorentz	0.98284		Lorentz	0.94533		Lorentz	0.99030
Gauss	0.98830		Gauss	0.9628		Gauss	0.955021		Gauss	0.97246
Mix	0.99097		Mix	0.985108		Mix	0.957033		Mix	0.99249
Dispesion	0.9914		Dispesion	0.993095		Dispesion	0.96291		Dispesion	0.98228
Rom Lorentz	0.99798		Rom Lorentz	0.989705		Rom Lorentz	0.98043		Rom Lorentz	0.993607
Rom Gauss	0.998425		Rom Gauss	0.993607		Rom Gauss	0.984865		Rom Gauss	0.996273
**SG400PtCalc used**			**SG400PtH$_2$red used**			**SG450PtCalc used**			**SG450PtH$_2$red used**	
	R			R			R			R
Lorentz	0.98669		Lorentz	0.99414		Lorentz	0.94280		Lorentz	0.99437
Gauss	0.98293		Gauss	0.96861		Gauss	0.91487		Gauss	0.97221
Mix	0.98875		Mix	0.99498		Mix	0.94280		Mix	0.99566
Dispesion	0.98855		Dispesion	0.996156		Dispesion	0.9470		Dispesion	0.996438
Rom Lorentz	0.995851		Rom Lorentz	0.996672		Rom Lorentz	0.953247		Rom Lorentz	0.98669
Rom Gauss	0.994131		Rom Gauss	0.998453		Rom Gauss	0.952231		Rom Gauss	0.997970

The ESR results on selected sol–gel based Pt-PtO$_x$/TiO$_2$ samples before and after the photocatalytic reaction are compared in Figure S8. The g values of the freshly prepared and used samples are slightly different. The line width of the reduced samples is significantly larger than that of the calcined ones, although an increase for the latter is evident during the photocatalytic reaction. The most likely cause of line broadening is the so-called g-strain (owing to small sample inhomogeneity). It may be noted that a correlation is apparent between the pre-annealing temperature of the sol–gel based support and the extent of the line broadening during the photocatalytic process.

In addition, the anisotropy of the g-values seems to be slightly more pronounced in the reduced samples. From the values of g, it is not possible to clearly determine the origin of the sharp line, although it is still very probable that the signal could be attributed to a single electron trapped in an oxygen vacancy [12].

The differing anisotropy and line widths suggest that the reductive or oxidative co-catalyst activation may result in the formation of vacancies with somewhat different environments. It is interesting to see that the line width difference between the recovered samples formed by H_2 treatment and calcination significantly decreases during the photocatalytic reaction which means that the nature of the defect sites became more similar after the photocatalytic action. This observation coincides with the XPS results, which also indicate that the surface state of all recovered sol–gel based photocatalysts is very similar. In turn, the similar Pt chemical states and the similar defect structure of the semiconductor indeed explain the similar catalytic behavior of the SG catalysts formed by H_2 reduction or calcination.

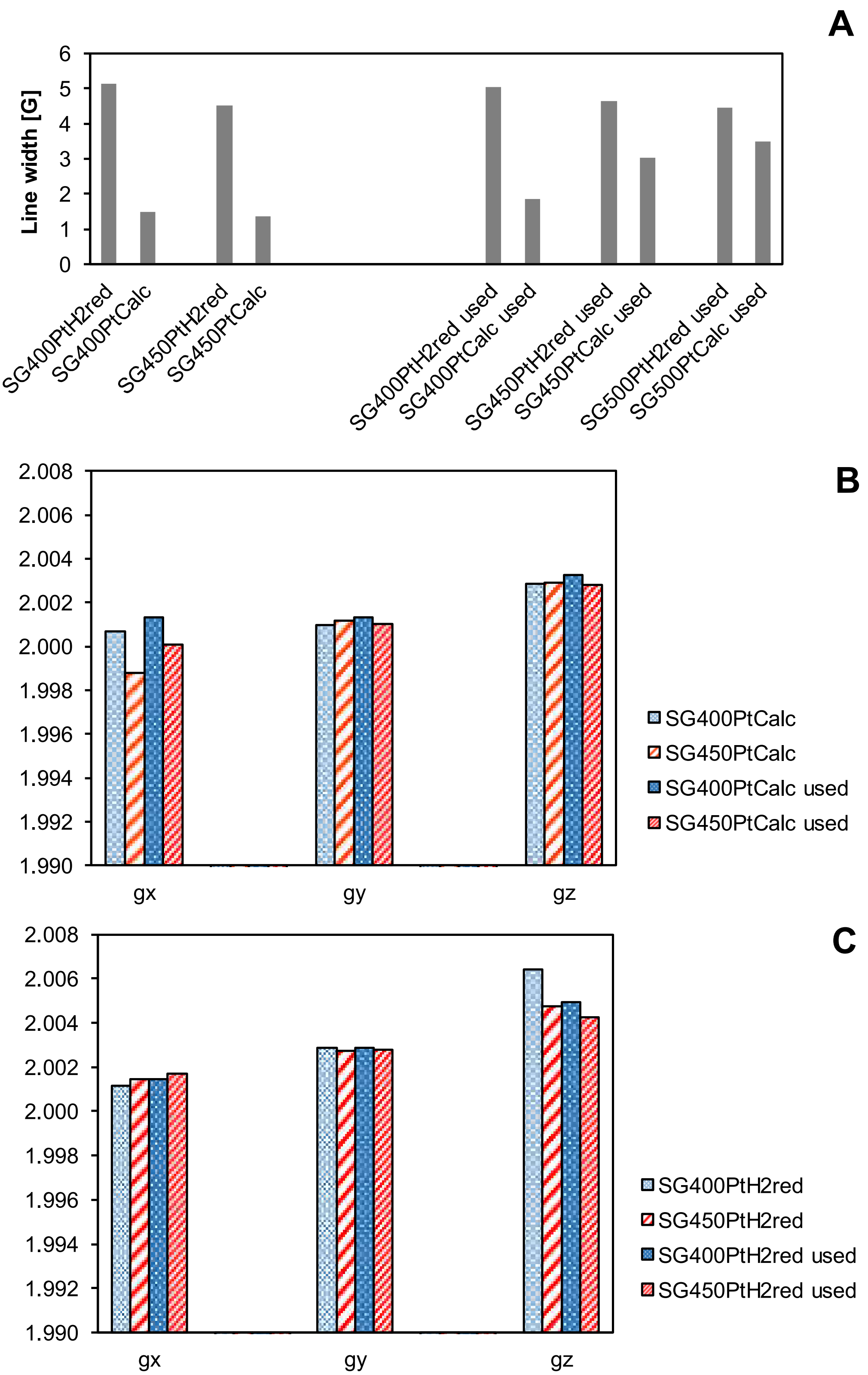

Figure S8. Comparison of electron spin resonance (ESR) results of selected sol–gel based fresh and recovered Pt-PtO_x/TiO_2 catalysts. A: line width; B: g values of samples from co-catalyst formation by calcination; C: g values of samples from co-catalyst formation by high-temperature H_2 treatment.

References

1. Moulder, J.F.; Stickle, W.F.; Sobol, P.E.; Bomben, K.D. *Handbook of X-Ray Photoelectron Spectroscopy*; Chastain, J., Eds.; Perkin-Elmer Corporation: Eden Prairie, MN, USA, 1992.
2. Wagner, C.D.; Naumkin, A.V.; Kraut Vass, A.; Allison, J.W.; Powell, C.J.; Rumble, C.J. NIST X-ray Photoelectron Spectroscopy Database, Version 3.4, National Institute of Standards and Technology: Gaithersburg, MD, USA, 2003.
3. CasaXPS Processing Software. Casa Software Ltd.: Teignmouth, UK. Available online: http://www.casaxps.com/ (accessed on 15 March 2008).
4. Mohai, M. XPS MultiQuant: Multimodel XPS quantification software. *Surf. Interface Anal.* **2004**, *36*, 828–832.
5. M. Mohai, " Multi-model X-ray photoelectron spectroscopy quantification program. Version 7.00.92, 2011 (available at the author's website without charge), http://aki.ttk.mta.hu/XMQpages/XMQhome.php (accessed on 05 February 2021).
6. Klein, G.A.; Meyrath, T. *Industrial color physics*, Springer: New York, NY, USA, 2010.
7. Landmann, M.; Rauls, E.; Schmidt, W.G. The electronic structure and optical response of rutile, anatase and brookite TiO_2. *J. Physics Condens. Matter* **2012**, *24*, 195503, doi:10.1088/0953-8984/24/19/195503.
8. Mistrik, J.; Kasap, S.; Ruda, H.E.; Koughia, C.; Singh, J. Optical Properties of Electronic Materials: Fundamentals and Characterization. In *Springer Handbook of Electronic and Photonic Materials*. Kasap, S., Capper, P. Eds. Springer: Cham, Switzerland.
9. Braginsky, L.; Shklover, V. Light absorption in TiO_2 nanoparticles. In *The european physical journal D*. Springer: Berlin, Heidelberg, Germany, 1999.
10. Studenyak, I., , Kranjcec, M., Kurik, M., Urbach rule in solid state physics. *Int. J. Opt. Appl.* **2014**, 4, 76-83.
11. Tálas, E.; Pászti, Z.; Korecz, L.; Domján, A.; Németh, P.; Szíjjártó, G.P.; Mihály, J.; Tompos, A. PtO_x-SnO_x-TiO_2 catalyst system for methanol photocatalytic reforming: Influence of cocatalysts on the hydrogen production. *Catal. Today* **2018**, *306*, 71–80.
12. Liu, H.; Ma, H.; Li, X.; Li, W.; Wu, M.; Bao, X. The enhancement of TiO_2 photocatalytic activity by hydrogen thermal treatment. *Chemosphere* **2003**, *50*, 39–46, doi:10.1016/s0045-6535(02)00486-1.
